# The Synergic Effect of *h*-MoO_3_, α-MoO_3_, and β-MoO_3_ Phase Mixture as a Solid Catalyst to Obtain Methyl Oleate

**DOI:** 10.1021/acsami.4c08804

**Published:** 2024-10-29

**Authors:** Gabrielle Sophie Medeiros Leão, Marcos Daniel Silva Ribeiro, Rubens Lucas de Freitas Filho, Libertalamar Bilhalva Saraiva, Ramón R. Peña-Garcia, Ana Paula de Carvalho Teixeira, Rochel Montero Lago, Flávio Augusto Freitas, Silma de Sá Barros, Sérgio Duvoisin Junior, Yurimiler Leyet Ruiz, Francisco Xavier Nobre

**Affiliations:** † Departamento de Química, Meio Ambiente e Alimentos (DQA), Grupo de Recursos Energéticos e Nanomateriais (GREEN Group), Instituto Federal de Educação, Ciência e Tecnologia do Amazonas, Campus Manaus Centro, Manaus 69020-120, AM, Brazil; ‡ Departamento de Química, ICEx, Universidade Federal de Minas Gerais, UFMG, Belo Horizonte, MG 31270-901, Brazil; § Universidade Federal Rural de Pernambuco, Programa de Pós-Graduação em Engenharia Física, UFPE, Recife, PE 52171-900, Brazil; ∥ Núcleo de Materiais e Energia − Centro de Bionegócios da Amazônia, Av. Gov. Danilo de Matos Areosa, 160 - Distrito Industrial I, Manaus, AM 69075-351, Brazil; ⊥ Programa de Pós-graduação em Engenharia de materiais, Escola de Engenharia de Lorena, Universidade de São Paulo, Estrada Municipal Chiquito de Aquino, n° 1000 − Mondesir, Lorena, SP 12612-550, Brazil; # Curso de Engenharia Química, Universidade do Estado do Amazonas, Escola Superior de Tecnologia, Av. Darcy Vagas, 1200, Parque Dez de Novembro, Manaus, AM 69050-020, Brazil; ∇ Departamento de Engenharia de Materiais, Laboratório de Processamento de Materiais Tecnológicos (LPMaT), Universidade Federal do Amazonas, Instituto de Ciências Exatas, Rua Av. General Rodrigo Otávio Jordão Ramos, 1200, Coroado I, Manaus 69067-005, Brazil

**Keywords:** Catalysis, molybdenum oxide, methyl oleate, synergism

## Abstract

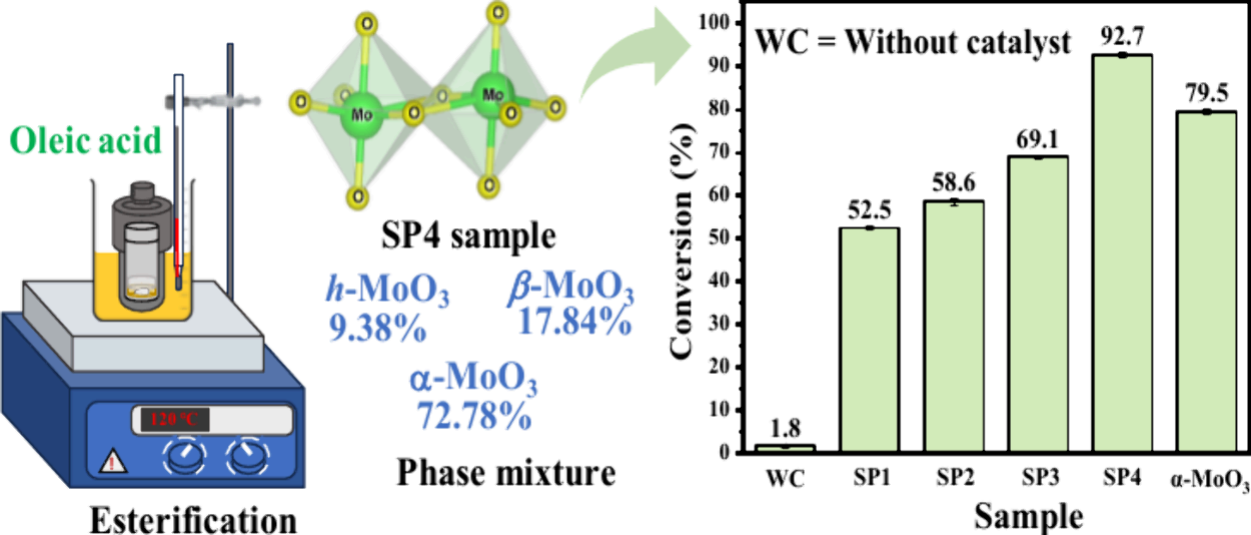

Extensive research in the last few decades has conclusively demonstrated the significant influence of experimental conditions, surfactants, and synthesis methods on semiconductors’ properties in technological applications. Therefore, in this study, the synthesis of molybdenum oxide (MoO_3_) was reported by the addition of 2.5 (MoO_3__2.5), 5 (MoO_3__5), 7.5 (MoO_3__7.5), and 10 mL (MoO_3__10) of nitric acid, obtaining the respective concentrations of 0.6, 1.10, 1.6, and 0.6 mol L^–1^. In this study, all samples were synthesized by the hydrothermal method at 160 °C for 6 h. The materials obtained were structurally characterized by X-ray diffraction (XRD) and structural Rietveld refinement, Raman spectroscopy, and infrared spectroscopy (FTIR), confirming the presence of all crystallographic planes and bands associated with active modes for the pure hexagonal phase (*h*-MoO_3_) when the solution’s concentration was 0.6 mol L^–1^ of nitric acid. For concentrations of 1.10, 1.60, and 2.10 mol L^–1^, the presence of crystallographic planes and active modes associated with the formation of mixtures of molybdenum oxide polymorphs was confirmed, in this case, the orthorhombic, monoclinic, and hexagonal phases. X-ray photoelectron spectroscopy reveals the occurrence of the states Mo^4+^, Mo^5+^, and Mo^6+^, which confirm the predominance of the acid Lewis sites, corroborating the analysis by adsorption of pyridine followed by characterization by infrared spectroscopy. The images collected by scanning electron microscopy confirmed the information presented in the structural characterization, where microcrystals with hexagonal morphology were obtained for the MoO_3__2.5 sample. In contrast, the MoO_3__5, MoO_3__7.5, and MoO_3__10 samples exhibited hexagonal and rod-shaped microcrystals, where the latter morphology is characteristic of the orthorhombic phase. The catalytic tests carried out in the conversion of oleic acid into methyl oleate, using the synthesized samples as a heterogeneous catalyst, resulted in conversion percentages of 52.5, 58.6, 69.1, and 97.2% applying the samples MoO_3__2.5, MoO_3__5, MoO_3__7.5, and MoO_3__10, respectively. The optimization of the catalytic tests with the MoO_3__10 sample revealed that the conversion of oleic acid into methyl oleate is a thermodynamically favorable process, with a variation in the Gibbs free energy between −67.3 kJ mol^–1^ and 83.4 kJ mol^–1^ as also, the energy value of activation of 24.6 kJ mol^–1^, for the temperature range from 80 to 140 °C, that is, from 353.15 to 413.15 K, respectively. Meanwhile, the catalyst reuse tests resulted in percentages greater than 85%, even after the ninth catalytic cycle. Therefore, the expressive catalytic performance of the mixture of *h*-MoO_3_ and α-MoO_3_ (MoO_3__10) phases is confirmed, associated with the synergistic effect, mainly due to the increase in the surface area and available Lewis sites of these phases.

## 1 Introduction

Current population growth and, consequently, growing global demand for energy, has been one of the main factors associated with increased dependence on natural resources, especially those of a nonrenewable nature, as raw material in the production of fuels.^1−3^ In this context, oil and derivatives are the main leading greenhouse gases, among others; the carbon dioxide (CO_2_), sulfur compounds (SO_2_), fluorides, and various types of metals, in addition to compromising human health, intensify climate change and the increase in natural disasters.^4−6^

In recent decades, several studies have sought to mitigate the environmental impacts associated with the unrestrained use of conventional fuels, conducting studies that reinforce the need for the total or partial replacement of these substances with alternative and sustainable sources. Therefore, using biomass to produce biofuels has been one of the efficient measures in obtaining clean energy and using residual biomass sources, such as fats and waste oils.^3,7,8^ In this context, biodiesel emerges as a promising alternative due to its renewable origin, nontoxic characteristics, and free of sulfur and aromatics, standing out for presenting considerably lower greenhouse gas emissions than that of common diesel.^9−11^

Biodiesel comprises fatty acid esters produced from the esterification or transesterification reactions of vegetable oils or animal fat with short-chain alcohols, such as methyl or ethyl. In the esterification or transesterification processes, the product has no elements such as nitrogen, sulfur, or lead, making the product have less impact on the environment when compared to petroleum diesel.^1,12,13^ This biofuel has similar physical-chemical properties to petroleum-based diesel, making it possible to mix both in different proportions.^14−16^ However, obtaining biodiesel is a process that suffers from the reversibility of the chemical reaction, therefore requiring catalytic species to ensure maximum conversion and reduction of the synthesis time.

Besides increasing the yield of the product of interest, homogeneous, heterogeneous, or enzymatic catalysts are commonly employed to reduce the effect of reversibility and reaction time.^17−21^ However, due to these materials’ corrosive, recalcitrant effect and low cost-benefit ratio, new catalysts, especially heterogeneous ones, have been investigated, increasing the options of promising and economically viable materials for this purpose. Among the classes of heterogeneous catalytic materials used in biofuel synthesis, we can find calcium oxide,^22^ zeolites,^12^ ionic liquids,^11^ hydroxyapatites,^23^ mesoporous silica,^24^ Metal–Organic-Frameworks (MOFs),^25^ semiconductor oxides,^26^ and waste materials, such as ash,^27^ biochar,^28^ and alkaline battery paste.^1^

Among the semiconductors extensively studied for various technological applications, it is possible to highlight molybdenum oxide (MoO_3_), a semiconductor known for exhibiting excellent semiconductor,^29^ optics,^30^ magnetic,^31^ catalytic,^32^ gas sensor,^33^ adsorptive,^34^ and electrochemical properties.^35^ Molybdenum oxide naturally exhibits three defined stoichiometric structures, commonly known as alpha, beta, and monoclinic phases.^36^ The alpha phase (α-MoO_3_) is the thermodynamically stable phase, which crystallizes in an orthorhombic structure with a space group of *Pbnm* and lattice parameters *a* = 3.9628(7) Å, *b* = 13.855(3), Å, *c* = 3.6964(6) Å, with four formulas per unit cell, Z = 4.^35^ On the other hand, the beta phase (β-MoO_3_) has a hexagonal structure (*P63/m*), which displays the lattice parameters *a* = *b* = 10.5680 Å and *c* = 3.7260 Å and two formulas per unit cell, Z = 2.^37^ In comparison, the monoclinic phase has a space group *P121/m1*, with lattice parameters *a* = 3.954(1) Å, *b* = 3.687(2) Å, and *c* = 7.095(4) Å and two formulas per unit cell Z = 2.^38^

In the study carried out by Silva et al.,^39^ the composite formed by the heterojunction between molybdenum oxide and reduced graphene oxide showed excellent catalytic properties in esterification and transesterification reactions of waste cooking oil under different experimental conditions, obtaining conversion percentages into methyl esters greater than 95.6%. On the other hand, Figueiredo et al.^40^ studied the catalytic performance of the heterojunction between molybdenum oxide and SBA-15 zeolite, which was found to obtain yields greater than 95% in the transesterification of soybean oil at a temperature of 150 °C and rotation of 500 rpm, adopting a factorial design 2^3^, with time and catalyst dosage as variables. The effect of heat treatment on the calcination of molybdenum trioxide was also investigated by Pinto et al.,^41^ correlating structural and morphological properties with catalytic performance in converting different vegetable oils into biofuels, resulting in the best performance for the sample obtained under heat treatment at 600 °C. However, the experimental conditions adopted in synthesizing the catalyst directly imply its cost-benefit due to the adoption of high temperatures in the heat treatment process.

Several methodologies are used to obtain molybdenum oxide; however, the characteristics and properties exhibited by these materials differ completely, making it possible to increase or decrease the properties of interest. Therefore, the literature has reported success in synthesizing polymorphs of molybdenum oxide using the conventional hydrothermal route,^42^ hydrothermal-assisted heat treatment,^41^ microwave-assisted hydrothermal,^37^ combustion,^14^ sonochemistry,^43^ solid-state synthesis,^44^ and chemical coprecipitation method.^45^ Although several scientific publications have been presented over the last few decades for MoO_3_ polymorphs, little has been explored for the mixture of phases of its polymorphs, considering that the mixture of semiconductors can lead to a synergistic effect, as well as antagonistic, mainly in the field of catalytic applications.

Routray et al.^46^ present the study of the synergistic properties of the combination between molybdenum oxide and iron molybdate −Fe_2_(MoO_4_)_3_ in the selective oxidation of methanol to formaldehyde, reaching high selectivity (≈80%), for the proportion Mo/Fe = 2.0. In the study carried out by Vibavakumar et al.,^47^ the synergistic effect on the electrochemical properties of the MoO_3_/MoS_2_ mixture was investigated, confirming the increase in the density of active sites capable of promoting the oxidation/reduction of the pair I_3_/3I^–^, obtaining current density (*J*_sc_) and efficiency (η,%) equal to 11.2 mA/cm^2^ and 3.9%, respectively; furthermore, in the study reported by Jada et al.,^48^ the synergistic effect between MoO_3_ and titanium dioxide (TiO_2_) was confirmed against the microbiological inhibition of strains of the bacteria *Escherichia coli*, confirming the increase in photoluminescent and antimicrobial properties for the sample containing 5% supported MoO_3_ in TiO_2_.

Considering the discussed facts, this article aims to investigate the catalytic properties of the MoO_3_ compound, especially the synergistic effect between the hexagonal, monoclinic, and orthorhombic phases obtained by the hydrothermal method at different synthesis temperatures. Furthermore, after being characterized by different analytical techniques, the materials were studied in esterification reactions, acting as a heterogeneous catalyst converting oleic acid into methyl oleate under different experimental conditions.

## 2 Materials and Method

### 2.1 Synthesis of Molybdenum Trioxide Samples −MoO_3_

**Figure 1:**
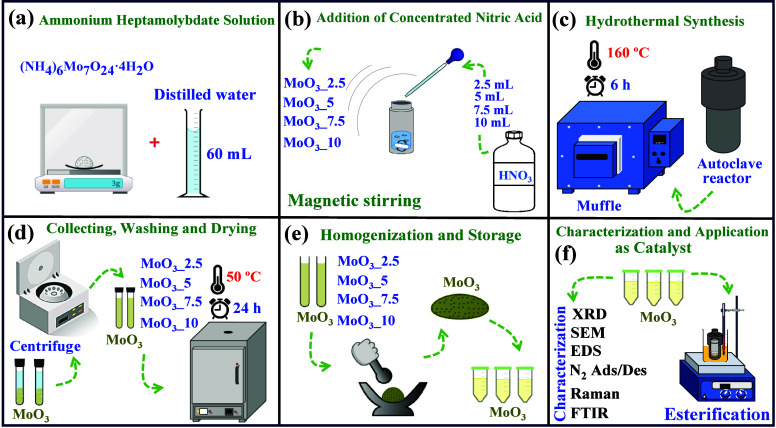
Schematic representation for (a) preparation of ammonium heptamolybdate solution, (b) addition of concentrated nitric acid, (c) hydrothermal synthesis, (d) collecting, washing, and drying the samples, (e) milling and storage of samples, and (f) characterization and application of all samples as solid catalyst in esterification of oleic acid.

In the synthesis of MoO_3_ microcrystals, all reagents were of analytical grade and used without further purity. Therefore, the MoO_3_ samples were prepared using the hydrothermal method following the steps mentioned by Chithambararaj and Bose et al.^49^ with adaptations, as seen in the steps shown in Figure 1(a-f).

Initially, 3g of ammonium heptamolybdate heptahydrate [(NH_4_)_6_Mo_7_O_24_·4H_2_O, Sigma-Aldrich, purity ≥99.0%] was added to 60 mL of distilled water under constant magnetic stirring. After the complete solubilization, 2.5, 5.0, 7.5, and 10.0 mL of concentrated nitric acid (HNO_3_, Sigma-Aldrich, purity >65%) were added drop-by-drop to the reaction medium, which remained under constant magnetic stirring. In this case, we obtained the samples MoO_3__2.5 (0.6 mol L^–1^), MoO_3__5 (1.10 mol L^–1^), MoO_3__7.5 (1.60 mol L^–1^), and MoO_3__10 (2.10 mol L^–1^), respectively.

For each case, the suspension obtained remained for 10 min under constant magnetic stirring, followed by transfer of the solution to an autoclave hydrothermal reactor (100 mL capacity), which was heated in a muffle furnace at 160 °C for 6 h. The precipitate (samples) was collected by centrifugation (4000 rpm for 5 min), washed several times with distilled water, and dried in an oven at 50 °C for 24 h. The materials were stored for characterization and subsequent catalytic tests.

### 2.2 Characterization

#### 2.2.1 X-ray Diffraction

The XRD patterns were obtained using a Shimadzu X-ray diffractometer, XRD-7000, using copper anode as an X-ray source (Cukα = 1.5406 Å), where the diffraction data were recorded in the 2θ range from 5° to 100°, at current and voltage of 10 mA and 30 kV, respectively. The structural analysis of phase composition (lattices parameters (*a*, *b*, *c*, α, β, γ) of the unit cell and atomic position (*x*, *y*, *z*), background, and crystallite size) was performed in detail using the Rietveld refinement adopting the software FullProf suite for Windows, version 2024, July.

#### 2.2.2 Vibration Raman and FTIR Spectroscopy

The vibrational Fourier transformed infrared spectrum (FTIR) of the samples was collected using a Bruker spectrometer, VERTEX 70 V, coupled with an Attenuated Total Reflectance (ATR) module, operating with a diamond crystal. The FTIR spectrum for each sample was collected using 32 scans with a resolution of 4 cm^–1^ in a vacuum transmittance module in the spectral range of 400 to 4000 cm^–1^. On the other hand, the study of active vibrational modes for molybdenum oxide (MoO_3_) structures was carried out using Raman spectroscopy. In this case, using a Bruker confocal Raman microscope, SENTERRA, with a laser wavelength of 532 nm (green laser), where the spectra were collected in the range of 50 to 1100 cm^–1^, with a resolution of 2 cm^–1^, 10 co-additions, and integration time of 10 s^–1^.

#### 2.2.3 N_2_ Adsorption/Desorption

Nitrogen adsorption/desorption analyses were carried out using Autosorb iQ equipment (Quantachrome Instruments, USA) at −196 °C in the relative pressure range of 0.005–1.0, using 300 mg of sample, which was previously degassed at 140 °C for 12 h under vacuum conditions. The surface area of each material was estimated by the BET method (Brunauer, Emmett, Teller), the pore size distribution was estimated by the BJH method (Barrett–Joyner–Halenda), and the total pore volume was obtained in the pressure range of relative 0.95. Experimental data and data processing were acquired using ASiQwin software, version 5.21.

#### 2.2.4 Scanning Electron Microscopy and Energy Dispersive X-ray (SEM-EDX)

Scanning electron microscopy (SEM) was performed by using an FEI Company microscope (Quanta FEG 250) to obtain the micrographs. An acceleration voltage of 200 to 30 kV, besides beam current >100 nA, was used, with a resolution of 1.6 nm at 3 kV in low vacuum and magnification between 12 and 1,000,000. All images were captured using the secondary electron detector to collect semiquantitative information by applying energy dispersive X-ray (EDX).

#### 2.2.5 X-ray Photoelectron Spectroscopy (XPS)

The semiquantitative and energy states analysis of molybdenum (Mo), nitrogen (N), and oxygen (O) were carried out by high-resolution XPS spectroscopy using a Thermo ScientificTM K-AlphaTM+ (Thermo Fisher Scientific, Waltham, MA, USA) spectrometer equipped with an aluminum monochromator, an X-ray energy of 1487 eV (Al Kα), and pass energy of 50 eV. The equipment was programmed to provide a spot size of 300 μm, while the spectra were acquired with an energy step of 0.1 eV and an acquisition time of 50 ms.

#### 2.6.6 Qualitative Determination of Lewis and Brønsted Sites by Adsorption/Desorption of Pyridine

The samples were dried at 105 °C for 24 h to remove moisture. A portion of each dried sample was stored for control analysis, while the remaining portion was transferred to a hermetically sealed container. This container was connected to a system saturated with pyridine vapor, and the samples were exposed to pyridine in a controlled atmosphere for 24 h. Following the pyridine exposure, both pyridine-treated and untreated samples were immediately subjected to a Bruker Fourier-transform infrared equipment by attenuated total reflectance (ATR) approach in the wavenumber range of 1700 to 1400 cm^–1^ (32 scans and resolution of 4 cm^–1^) to evaluate the surface chemistry and potential functional group interactions.

### 2.3 Catalytic Experiments

The esterification of oleic acid [CH_3_(CH_2_)_7_CH=CH(CH_2_)_7_COOH, Sigma-Aldrich, purity = 90%] was carried out in an autoclave reactor (25 mL capacity), utilizing an oil bath and constant magnetic stirring at 650 rpm. The conditions initially adopted to investigate the catalytic performance of the synthesized samples were a reaction time of 3 h; temperature of 120 °C; oleic acid/methanol molar ratio (CH_3_OH, Sigma-Aldrich, purity ≥99.98%) of 1:10; and catalyst dosage of 5% (m/m) concerning the mass of oleic acid.

According to Reis et al., the yield in the oleic acid esterification reaction was quantified.^50^ To this end, approximately 0.1 g of each sample was weighed in an Erlenmeyer flask (50 mL capacity), together with 20 mL of ethyl alcohol (Sigma-Aldrich, purity >92.8%), previously neutralized by 0.1 mol L^–1^ NaOH.

The samples were titrated in triplicate with a standardized solution of 0.1 mol/L NaOH and 2 drops of 1% phenolphthalein. The same procedure was performed using oleic acid as a blank or standard. With this, the acidity index was calculated using eq 1:
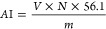
1where *V* is the volume of NaOH solution used in the titration in milliliters (mL), *N* is the concentration of the titrant solution, *m* is the mass of the sample weighed in grams, and 56.1 is the conversion constant to obtain the index of acidity in milligrams of potassium hydroxide per gram of sample (mg KOH/g). The catalysis conversion (%R) was determined based on the acidity of oleic acid (blank) and the samples, as reported by Tang and Niu^51^ available in eq 2:
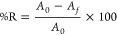
2where *A*_0_ corresponds to the acidity of oleic acid, and *A*_*f*_, to the acidity of the methyl oleate sample.

For all cases, the product from each catalytic experiment was separated by centrifugation, where the residual methanol was collected by using a rotary evaporator and a heating bath with digital temperature control at a rotation speed of 75 rpm and a temperature of 65 °C.

The optimization of catalytic performance was carried out by investigating the parameters: reaction time (0.5, 1, 3, and 5 h); temperature (80, 100, 120, and 140 °C); oleic acid/methanol molar ratio (1:5, 1:10, 1:15, and 1:20); and catalyst dosage (2.5, 5, 7.5, and 10%, m/m). After optimization of the parameters, the stability and reuse of the catalyst in 9 (nine) consecutive cycles was investigated. For these purposes, the catalyst was recovered by centrifugation, washed twice with hexane and twice with distilled water, and dried in an oven for 24 h at 80 °C.

## 3 Results and Discussion

### 3.1 X-ray Diffraction and Structural Rietveld Refinement

**Figure 2:**
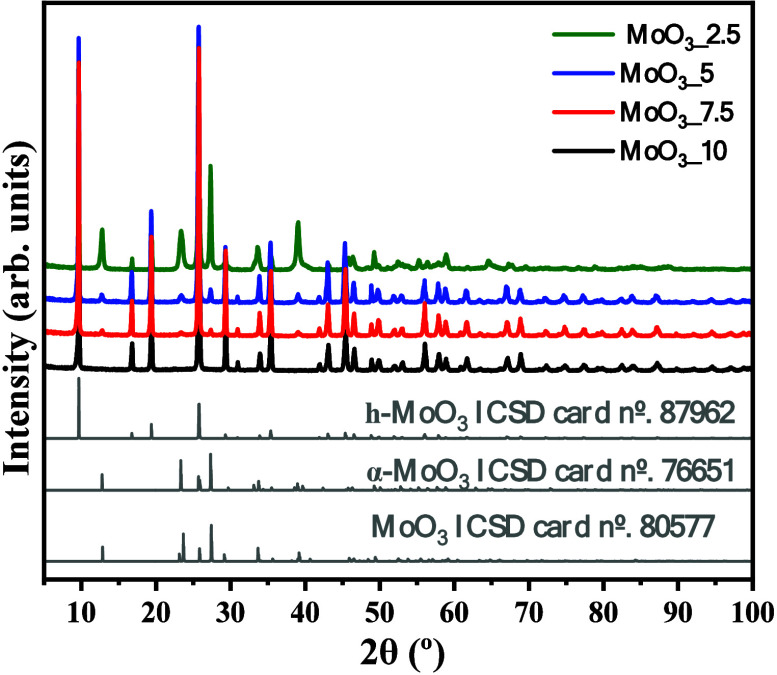
XRD pattern of MoO_3__2.5, MoO_3__5, MoO_3__7.5, and MoO_3__10 samples. For comparison, the XRD diffraction pattern of ICSD card Nos. 87962 (*h*-MoO_3_), 76651 (α-MoO_3_), and 80577 (β-MoO_3_) was inserted.

The structural characterization of the synthesized samples was initially conducted by X-ray diffraction (XRD), as shown in Figure 2. The intensity and profile of the diffraction peaks indicate the formation of materials with a high degree of crystallinity and short- and long-range organization, characteristic of materials obtained via hydrothermal synthesis; in addition, there are clear modifications that suggest the structural transition and consequent mixture of phases for the materials obtained. Indexing the diffraction patterns revealed the obtainment of the hexagonal phase with lattice parameters *a* = *b* = 10,5680 Å and *c* = 3,7260 Å, space group of *P63/m*, and two formulas per unit cell (Z = 2), for molybdenum trioxide synthesized at a concentration of nitric acid at 0.6 mol L^–1^. All crystallographic planes identified in the 2θ intervals from 5 to 100° agree with the crystallographic information on the Inorganic Crystal Structure Database card No. 35076, where peaks of high crystallinity identified at 2θ = 9.8° and 25.7° correspond to the crystallographic planes (100) and (210)/(101), respectively.

It is noted that the increase in the concentration of nitric acid in the reaction medium, in this case, 1.10 and 1.60 mol L^–1^, resulted in the emergence and gradual increase in the intensity of diffraction peaks, mainly those located at 2θ = 12.8°, 23.5°, 27.3°, and 38.9°. These are associated with the crystallographic planes (020), (110), (021), and (111), respectively. The crystallographic planes mentioned are characteristic of the orthorhombic structure for molybdenum trioxide, also known as the alpha phase (α-MoO_3_), which has space group *Pbnm* and lattice parameters of *a* = 3.9628 Å, *b* = 13.8550 Å, and *c* = 3.6964 Å and four formulas per unit cell, Z = 4. Moreover, the XRD peaks for sample MoO_3__10, which are obtained at an acid concentration of 2.0 mol L^–1^, suggest the occurrence of the monoclinic structure (*P121/m1*), β-MoO_3_, with lattice parameters *a* = 3.954(1) Å, *b* = 3.687(2) Å, and *c* = 7.095(4) Å and unit cell volume of 100.47(8) Å^3^, agreeing with the crystallographic information contained in the ICSD card No. 80577.

The phase mixture for molybdenum trioxide was also reported by Chithambararaj and Bose,^49^ using hydrothermal synthesis at temperatures of 90, 150, and 210 °C, processes for 12 h of reaction. Among other important information, the authors report obtaining the pure hexagonal phase for the sample processed at 90 °C. In comparison, at 150 °C, a mixture of phases was formed between the alpha and hexagonal phases, while at 210 °C, a pure orthorhombic phase was obtained.

**Figure 3:**
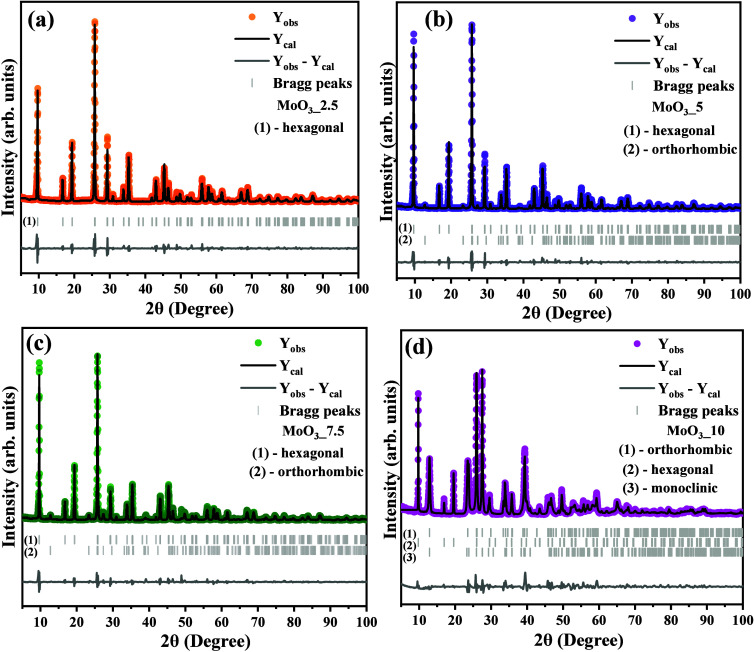
Structural Rietveld refinement plot for (a) MoO_3__2.5, (b) MoO_3__5, (b) MoO_3__7.5, and (d) MoO_3__10 samples.

The structural analysis by Rietveld refinement has been employed to obtain the phase composition (*Xr*) as well as the lattice parameters (*a*, *b*, *c*, α, β, and γ), atomic position (*x*, *y*, and *z*), occupation, and crystallite size (*D̅*_*hkl*_). The Rietveld refinement plot for all synthesized samples is shown in Figure 3(a-d).

Based on the plot profile of *Y*_obs_, *Y*_cal_, and the residual line (*Y*_obs_ – *Y*_cal_), it is confirmed that the computed and experimental data are in good agreement, where a minimum of differences of them is verified in the residual line. For this purpose, the crystallographic information from the ICSD card Nos. 35076, 62123, and 80577 (γ-MoO_3_), associated with the hexagonal, orthorhombic, and monoclinic structures, was adopted as input data in the analysis. For all cases, the quality of computed data was checked by qui-square (*χ2*) and values of R_profile (*R*_p_, *R*_wp_, and *R*_exp_), which indicate good and reliable results for computed data using the Pseudo-Voigt axial divergence asymmetry function. Furthermore, the occurrence of the hexagonal phase was confirmed for the *h*-MoO_3__2.5 sample. In contrast, for the *h*-MoO_3__5 and *h*-MoO_3__7.5 samples, there was a mixture of phases between the orthorhombic and hexagonal phase, while for the *h*-MoO_3__10 sample, the three polymorphs coexist; in this case, the emergence of the third polymorph (β-MoO_3_) emerges with a monoclinic structure.

As displayed in Table S1 (available in Supporting Information), the sample MoO_3__2.5 is composed basically of a hexagonal structure, which exhibits lattice parameters *a* = 10.575(3) Å, *b* = 10.575(3) Å, and *c* = 3.725(7) Å and unit cell volume of 360.85(02) Å^3^. Differently, for the samples MoO_3__5 and MoO_3__7.5, the percentage of the *h*-MoO_3_ polymorph was 97.96% and 95.71%, respectively. Moreover, the lattice parameters were, respectively, 10.574(3) and 10.578(1) Å for *a*-axis, 10.574(3) and 10.578(1) Å for *b*-axis, and for *c*-axis were: 3.725(7) and 361.03(9) Å. On the other hand, the orthorhombic structure is equal to 2.04% (MoO_3__5) and 4.29% (MoO_3__7.5), with lattice parameters of *a* = 13.855(9) Å and 13.867(3), *b* = 3.696(5) Å and 3.6949(4) Å, while the *c*-axis were 3.959(7) Å and 3.957(7) Å, corresponding to the MoO_3__5 and MoO_3__7.5 samples, respectively. Finally, for sample MoO_3_-10, the phase composition reached was 9.38% for *h*-MoO_3_, 72.78% for α-MoO_3_, and 17.84% for β-MoO_3_, that is, hexagonal, orthorhombic, and monoclinic structures, respectively. Therefore, for the MoO_3__10 sample, in addition to the emergence of the monoclinic phase, there was a predominance of the orthorhombic phase in its composition.

The results also show that increasing the concentration of nitric acid during the synthesis of molybdenum trioxide polymorphs not only changes the types of polymorphs formed but also leads to larger crystallite sizes in the hexagonal structure as calculated using the Scherrer equation (*D*_hkl_ = 0.91λ/(*B* cos θ). In this case, *B* is the full width at half-maximum of the characteristic diffraction peak obtained through the structural Rietveld refinement, while λ is the wavelength from the XRD equipment, which is adopted by the radiation, KαCu = 0.15406 nm, and θ is the diffraction angle characteristic of each crystallographic phase. Thus, it was observed three different plans: (210) for the hexagonal phase at 2θ = 25.9°, (021) for the orthorhombic phase at 2θ = 27.5°, and (011) associated with the monoclinic phase at 2θ = 27.4°. Therefore, resulting in crystallite sizes equal to 46 nm (MoO_3__2.5), 55 nm (MoO_3__5), 56 nm (MoO_3__7.5), and 77 nm (MoO_3__10) for the hexagonal phase. Differently, for the α-MoO_3_ phase, there was the opposite effect, where a decrease was noted from 22 nm (MoO_3__5) to 21 nm (MoO_3__7.5) and, finally, 20 nm for the MoO_3__10 sample. For the monoclinic phase, the determined crystallite size was 120 nm, present only in the MoO_3__10 sample.

Wu et al.^52^ report using different inorganic acids (HNO_3_, HCl, and H_2_SO_4_) to obtain molybdenum oxide polymorphs, using ammonium heptamolybdate as a synthesis precursor, adopting the microwave-assisted hydrothermal method. The authors reveal through the X-ray diffraction technique that when sulfuric acid was used, the predominantly orthorhombic phase was formed for all samples. On the other hand, when nitric and hydrochloric acids were used, the hexagonal phase predominated at temperatures equal to or lower than 170 °C, with the occurrence of phase mixing between the polymorphs *h*-MoO_3_ and α-MoO_3_ at a temperature of 200 °C. According to the authors, the phase conversion is due to the Brownian motion in the system under microwave heating, causing instability in the interaction of the NH_4_^+^ groups with the oxygens present in the terminal oxygens of the [MoO_6_] clusters.

Based on the results, it is believed that the increase in acidity by the addition of gradual volumes of nitric acid in obtaining the solutions used to prepare the samples (MoO_3__2.5, MoO_3__5, MoO_3__7.5, and MoO_3__10) led to the removal of theNH_4_^+^ groups from the structure, resulting in the instability of the *h*-MoO_3_ phase and the emergence of the α-MoO_3_ and β-MoO_3_ polymorphs. In addition, it is noted that the variations observed for the lattice parameters, unit cell volume, and crystallite size are directly related to the occurrence of oxygen vacancies and variations in the length and bond angle of the Mo–O groups in the structures.

Wu et al.^52^ report the use of different inorganic acids (HNO_3_, HCl, and H_2_SO_4_) to obtain molybdenum oxide microcrystals, in this case, the ammonium heptamolybdate as the precursor, adopting the microwave-assisted hydrothermal method. Therefore, X-ray diffraction analysis confirmed that the orthorhombic phase was predominantly formed for all samples prepared with sulfuric acid. On the other hand, when nitric and hydrochloric acids were used, the hexagonal phase is predominantly at temperatures ≤170 °C. However, phase mixing is confirmed for the polymorphs *h*-MoO_3_ and α-MoO_3_ at 200 °C. According to the authors, the phase conversion is due to the Brownian motion in the system under microwave heating, which caused instability in the interaction of the NH_4_^+^ groups with the oxygen present in the terminal oxygens of the [MoO_6_] clusters.

Based on the results obtained, it is believed that the increase in acidity by the addition of gradual volumes of nitric acid in obtaining the solutions used to prepare the samples MoO_3__2.5, MoO_3__5, MoO_3__7.5, and MoO_3__10 led to the removal of the NH_4_^+^ groups from the structure, resulting in the instability of the *h*-MoO_3_ phase, leading to the emergence of the α-MoO_3_ and β-MoO_3_ polymorphs. In addition, it is noted that the variations observed for the lattice parameters, unit cell volume, and crystallite size are directly related to the occurrence of oxygen vacancies and variations in the length and bond angles of the Mo–O groups in the structures.

### 3.2 Raman and Infrared Vibrational Spectroscopy

**Figure 4:**
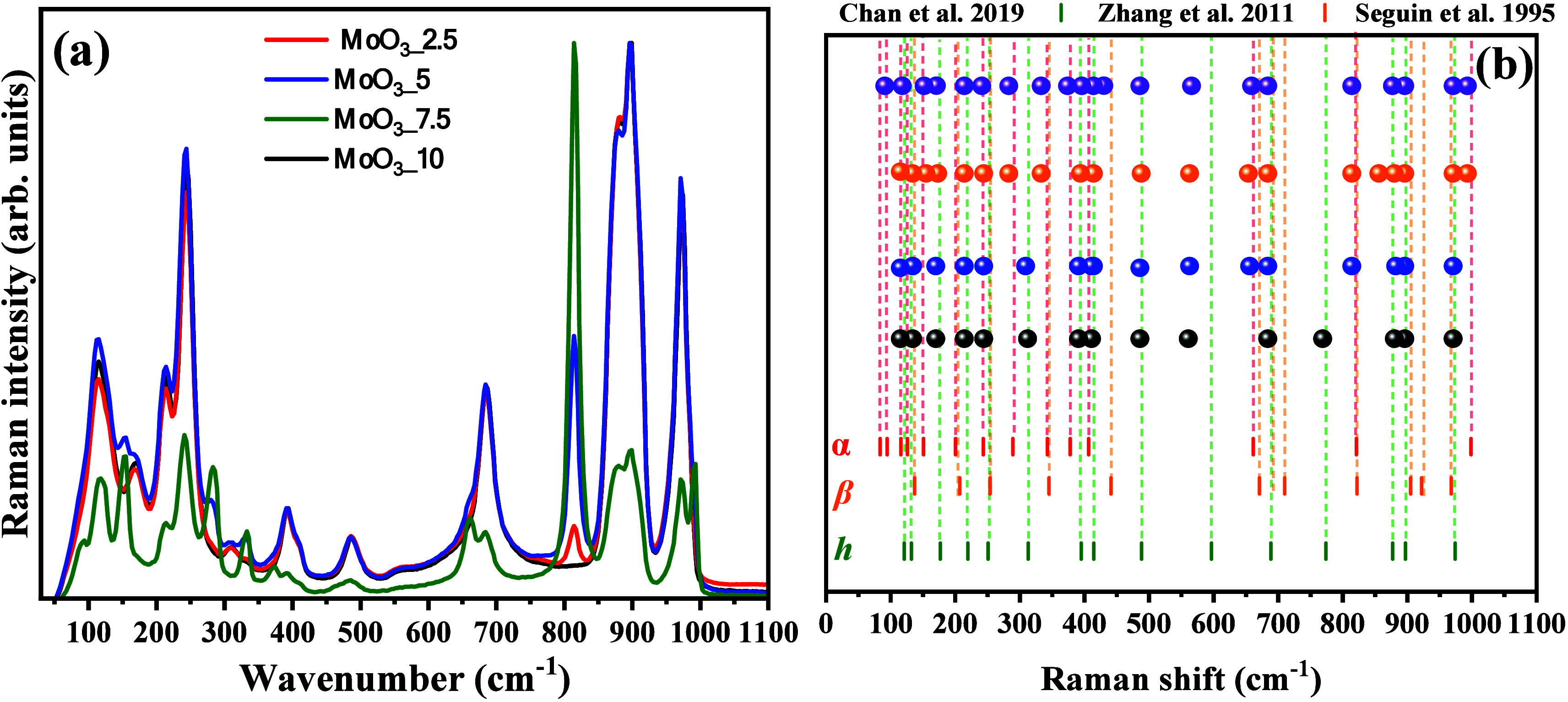
Vibrational Raman spectra of (a) MoO_3__2.5 (black balls), MoO_3__5 (blue balls), MoO_3__7.5 (orange balls), and MoO_3__10 (purple balls) samples, and (b) correlation of the band position of all synthesized samples with those reported by literature.^54,55,53^

To confirm the information obtained in the structural analysis by X-ray diffraction, vibrational Raman spectroscopy was used to characterize the materials, recording the vibrational modes associated with the respective crystalline structures from 50 to 1100 cm^–1^. In Figure 4(a,b), the spectra collected for samples MoO_3__2.5, MoO_3__5, MoO_3__7.5, and MoO_3__10 are graphically presented.

The literature reports that the hexagonal structure for space group molybdenum oxide *P63/m* displays sixty-nine optical modes and three acoustic modes, distributed in the irreducible formula for the point group *C*_6*h*_. However, only 20 active modes are expected in Raman spectroscopy, associated with symmetry elements A_g_, E_2g_, and E_1g_. On the other hand, Seguin et al.^53^ report, based on group theory, the existence of forty-five acoustic modes for the orthorhombic structure of MoO_3_ with space group *Pbnm*, associated with the elements of symmetry A_g_, B_1g_, B_2g_, and E_3g_, based on group theory, and 11 active modes in Raman spectrum associated with the space group of *P*21/*m* for the monoclinic structure.

As seen in the Raman vibrational spectrum (Figure 4a) collected for sample MoO_3__2.5, all vibrational modes present are characteristic of the hexagonal phase, in excellent agreement with the Raman spectrum presented by Zhang et al.^54^ They showed that the vibrational modes between 600 and 1000 are related to the vibrations of the octahedral symmetry [MoO_6_] clusters, while the vibrations between 200 and 400 cm^–1^ are characteristic of the torsional movements of the Mo–O bonds in the crystal lattice. Wavenumbers lower than 200 cm^–1^ are due to deformations of the bonds along the crystal lattice. In the spectra for MoO_3__5, MoO_3__7.5, and MoO_3__10 samples, it is noted that additional vibrational modes associated with the alpha phase were present, gradually increasing the intensity of the bands associated with the vibrational modes. Therefore, this confirms the information presented in the discussion about the collected X-ray patterns, where mixing between the hexagonal and alpha phases occurred for MoO_3__5, MoO_3__7.5, and MoO_3__10 samples.

Thus, the identified bands agree with the characteristics of the hexagonal phase in positions close to those identified for the MoO_3__2.5 sample, as can be observed in the indexing of the bands presented in Figure 4(b). Also, bands associated with the vibrational modes of the alpha phase were identified, where the bands of strong intensity in the range of 800 to 1000 cm^–1^ are due to the symmetric and asymmetric stretching of the Mo=O bonds, respectively. On the other hand, the bands between 300 and 700 cm^–1^ are characteristic of torsional and scissor movements of the O–Mo–O bonds, between 190 and 295 cm^–1^, the vibrational and rotational modes of the Mo=O bonds and rigid units of the [MoO_4_] clusters, and between 100 and 185 cm^–1^, are the translational modes of the [MoO_4_] groups along the crystal lattice.

As Figure 4b shows, all identified experimental band positions for active modes are in excellent agreement with the bands’ positions in the studies reported by Zhang et al.,^54^ Seguin et al.,^53^ and Chan et al.^55^

**Figure 5:**
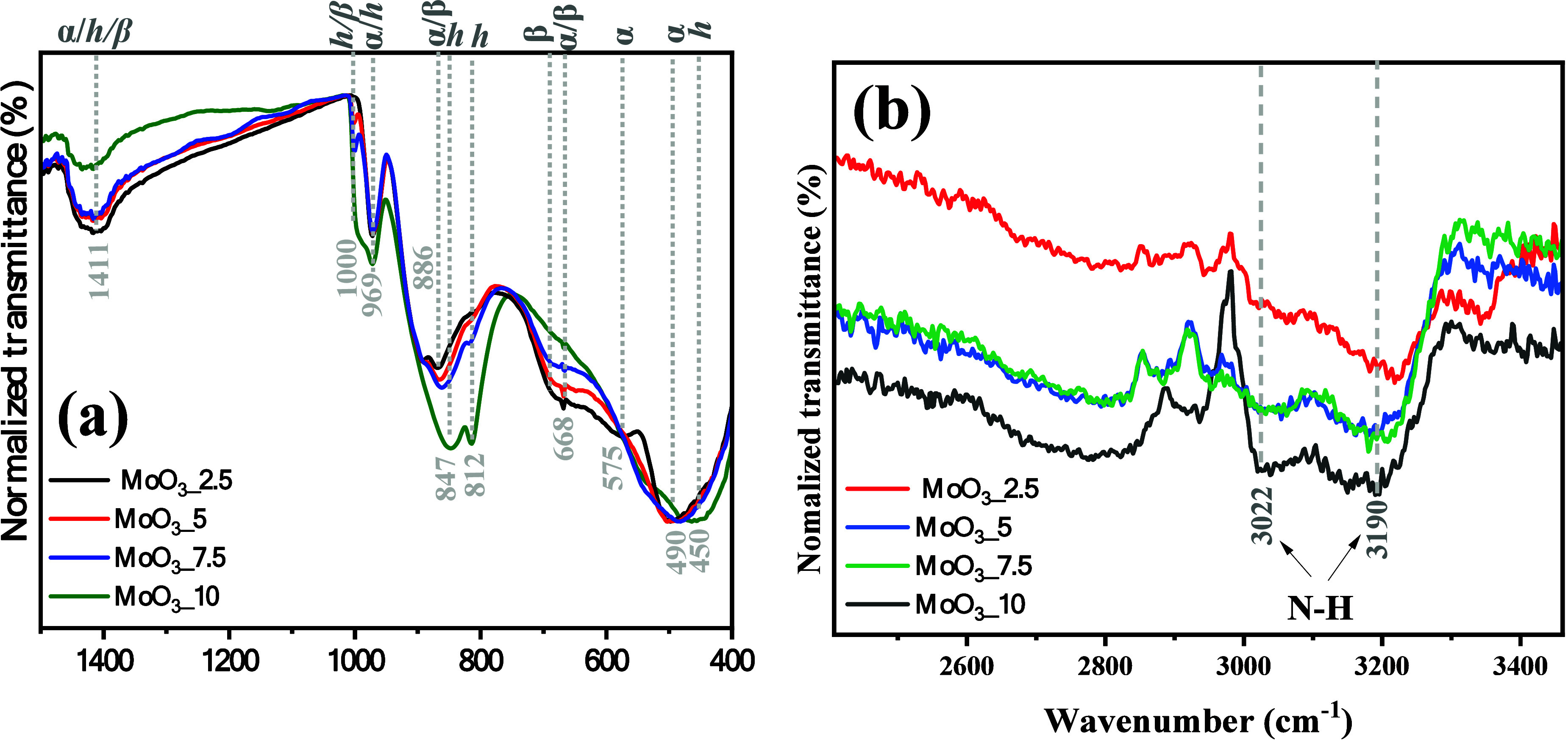
Vibrational FTIR spectra of (a) MoO_3__2.5, MoO_3__5, MoO_3__7.5, and MoO_3__10 samples, and (b) depicted interval from 2400 to 3400 cm^–1^.

The complementary analysis to Raman spectroscopy was carried out using Fourier transform infrared spectroscopy (FT-IR), as shown in Figure 5(a,b). Based on the results presented in Figure 5a, it is possible to confirm the presence of active vibrational modes for the hexagonal phase in the MoO_3__2.5 sample, corroborating the other analytical techniques presented in the previous discussions.

In this context, bands associated with Mo=O bond stretching were identified at wavenumbers 450, 812, and 451 cm^–1^. On the other hand, the bands at 575 and 847 cm^–1^ are due to the stretching movements of the O–Mo–O bonds in the octahedral symmetry [MoO_6_] clusters. Scissor-shaped movements for O–Mo–O bonds are associated with a low-intensity band centered at 668 cm^–1^. Vibrations of the O–H bonds of water molecules adsorbed in the structures were also identified at 1411 cm^–1^, overlapping the stretching of the N–H bonds from NH_4_^+^ ions present in the terminal groups of the [MoO_4_] clusters, characteristic of the hexagonal phase, mainly when synthesized from ammonium heptamolybdate, through hydrothermal processing.

The latter is confirmed by the presence of symmetric stretch bands at 3022 and 3190 cm^–1^, as shown in Figure 5(b), which suggests the occurrence of different frequencies for the stretches, which can be related to the proximity of Mo=O groups, which exhibit different electron densities than the O–Mo–O bonds. The vibrational modes identified in the spectra of MoO_3__5, MoO_3__7.5, and MoO_3__10 samples confirm the observations presented in vibrational Raman spectroscopy, in which the evident emergence and gradual increase in the intensity of the modes is noted at 490, 812, 847, and 969 cm^–1^, characteristic of the vibrations of the O–Mo–O and Mo=O bonds, present in the orthorhombic and monoclinic structures of MoO_3_.

### 3.3 Scanning Electron Microscopy and Energy Dispersive X-ray – SEM/EDX

**Figure 6:**
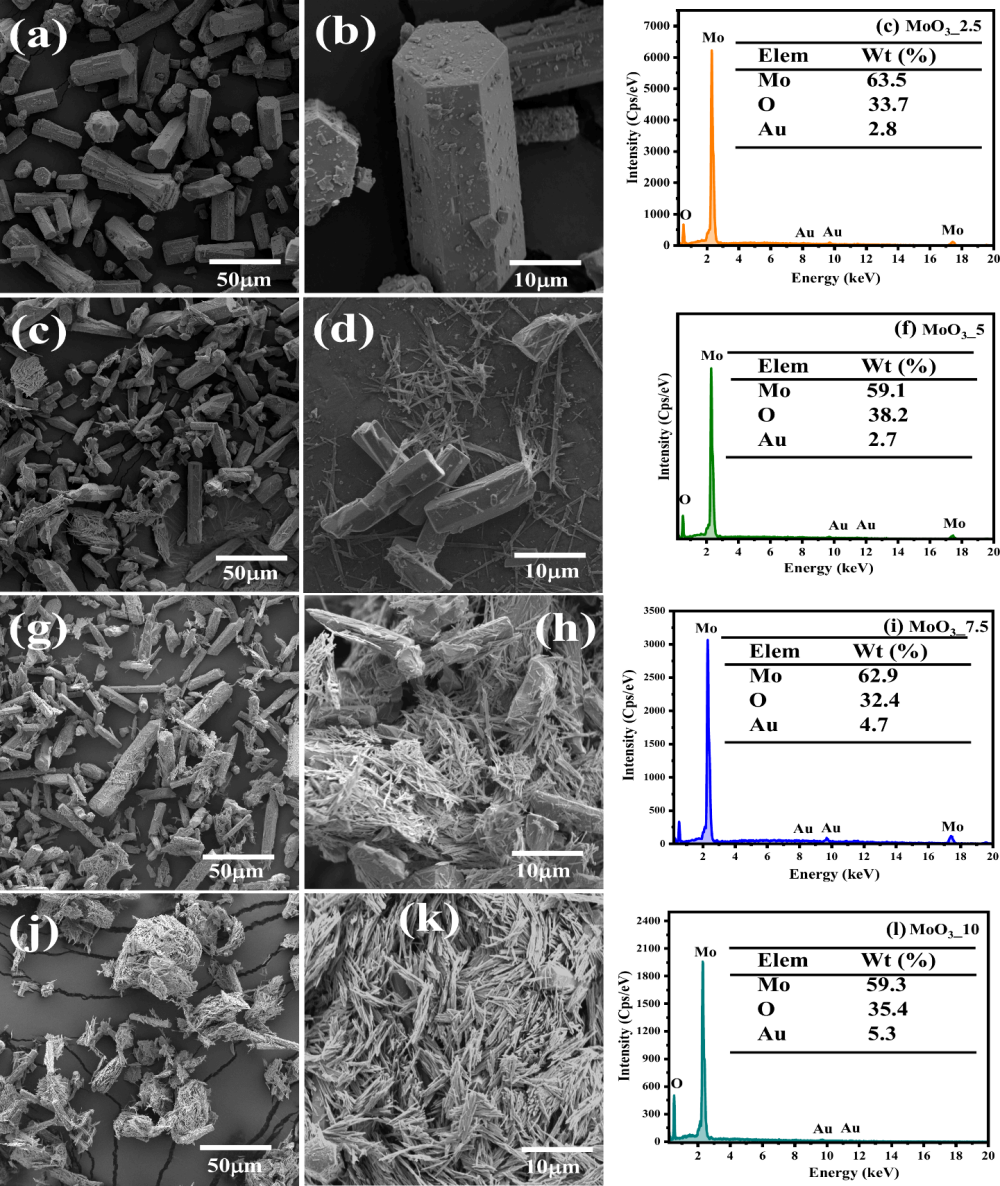
SEM images of (a, b) MoO_3__2.5, (d, e) MoO_3__5, (g, h) MoO_3__7.5, and (j, k) MoO_3__10 samples, and EDS spectrum of elements of (c) MoO_3__2.5, (f) MoO_3__5, (i) MoO_3__7.5, and (l) MoO_3__10 samples.

Semiquantitative textual and morphological analysis of the synthesized samples was carried out using scanning electron microscopy (SEM), as well as energy dispersive X-rays (EDX), as shown in Figure 6(a-l). Based on the information presented, it is possible to clearly distinguish the morphology of the crystals obtained, agreeing with the characteristic morphologies of the crystalline phases present in the structural (XRD) and vibrational (Raman and FITR) characterization. Thus, as predicted in the characterization techniques, sample MoO_3__2.5 presents the hexagonal phase as the only phase present for molybdenum oxide (*h*-MoO_3_), which is noticeable in the presence of microcrystals shaped like elongated rods (Figure 6a,b). In this study, the crystal length and weight for the MoO_3__2.5 sample are, respectively, 27.47 (±3.1) and 12.14 (±1.8) μm. Using the hydrothermal method, Vibavakumar et al.^47^ report the formation of rod-like microcrystals with length and width of 35 um and 7 um, respectively.

The microcrystals of the MoO_3__5 sample (Figures 6(d,e)) show the occurrence of rod-shaped crystals with sub-micrometric dimensions, characteristic of the orthorhombic and monoclinic polymorphs. They are anchored on the surface of microcrystals in hexagons, which is characteristic of the hexagonal phase. Moreover, there was a decrease in crystal length and width for 17.84 μm (±2.5) and 6.0 μm (±0.56), respectively. Submicron crystals are displayed for samples MoO_3__7.5 (Figure 5g,h) and MoO_3__10 (Figure 5j,k), where the arrangement of crystals with sub-micrometric dimensions and rod shape increases significantly. The crystal lengths for hexagon rod-like microcrystals are 21.8 μm (±1.2) and 22.9 μm (±1.3), MoO_3__7.5 and MoO_3__10 samples, respectively. At the same time, the respective crystal widths for hexagonal rod-like microcrystals are 6.23 μm (±0.8) and 6.32 μm (±0.6). Thus, this confirms the increase in the orthorhombic phase proportion in composition, as already predicted in the structural characterization by X-ray diffraction.

Semiquantitative analysis by EDS reveals the presence of the elements molybdenum (Mo), oxygen (O), and gold (Au), the latter being used to metalize the samples via sputtering. Therefore, the EDS spectra presented in Figures 6c, 6f, 6i, and 6l refer to MoO_3__2.5, MoO_3__5, MoO_3__7.5, and MoO_3__10 samples, respectively.

Based on these spectra, it is noted that the composition of the matrix indicates high purity of the synthesized samples, with no dispersive energy peaks associated with contaminants being identified. On the other hand, the percentage composition of the matrix elements indicates subtle variations in the amount of Mo (59.1–63.5%) and O (32.4–38.2%), mainly associated with the composition of phases present, which favors the occurrence of vacancies of oxygen, presence of NH_4_^+^ ions in the terminal [MoO_6_] clusters, and anchoring of the structures resulting in overlapping of the structures’ interfaces.

### 3.4 Surface Area and Pore Diameter by N_2_ Adsorption/Desorption

**Figure 7:**
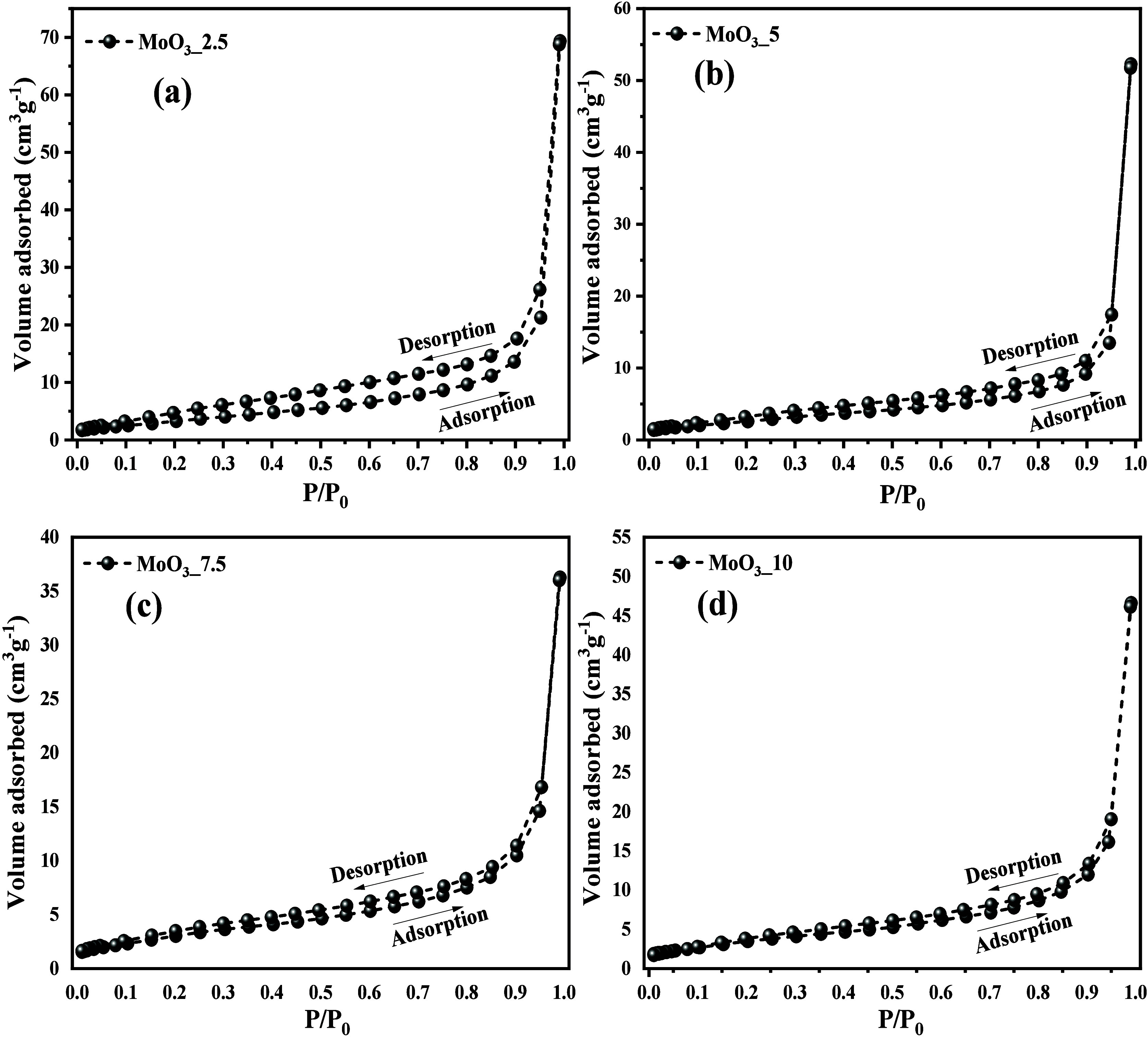
N_2_ adsorption/desorption hysteresis of (a) MoO_3__2.5, (b) MoO_3__5, (c) MoO_3__7.5, and (d) MoO_3__10 samples.

The textural analysis of the samples (pore diameter and volume and specific surface area) was carried out by the adsorption/desorption of nitrogen gas (N_2_) using the method developed by Brunauer, Emmett, and Teller (BET). As can be seen in Figure 7(a-d), the profile of N_2_ gas adsorption and desorption hysteresis obtained for the materials is type IV, with a type H1 curve, according to the classification of the International Union of Pure and Applied Chemistry (IUPAC) and literature consulted.^40^ In this case, it is characteristic of mesoporous materials.

It is possible to note that the adsorption capacity and the graphic profile for the samples were slightly different, resulting in the gradual increase in the adsorption of N_2_ molecules for MoO_3__2.5, MoO_3__5, MoO_3__7.5, and MoO_3__10 samples, obtaining surface area values of 1.69, 1.70, 2.72, and 10.2 m^2^/g, respectively. Meanwhile, the respective pore diameter values were 43.2, 40.0, 39.5, and 37.7 nm. Based on the information obtained, it is possible to suggest that the increase in the percentage of the orthorhombic phase for MoO_3_, which has crystal dimensions that are significantly lower than compared to the crystals obtained for the hexagonal phase, contributed significantly to the increase in surface area as well as reduction in pore diameter. These values are in agreement with those reported by Manivel et al.,^56^ who obtained the values of 0.43, 1.15, and 4.85 m^2^/g for molybdenum trioxide (*h*-MoO_3_) nanocrystals, synthesized by thermal decomposition, hydrothermal microwave, and sonochemical methods, respectively. Silva et al.^14^ also reported the synthesis of α-MoO_3_ but using the combustion method, obtaining materials with a surface area of 1.36 m^2^/g.

### 3.5 Pyridine Probe of Lewis and Brønsted Sites by Vibrational Infrared Spectroscopy

**Figure 8:**
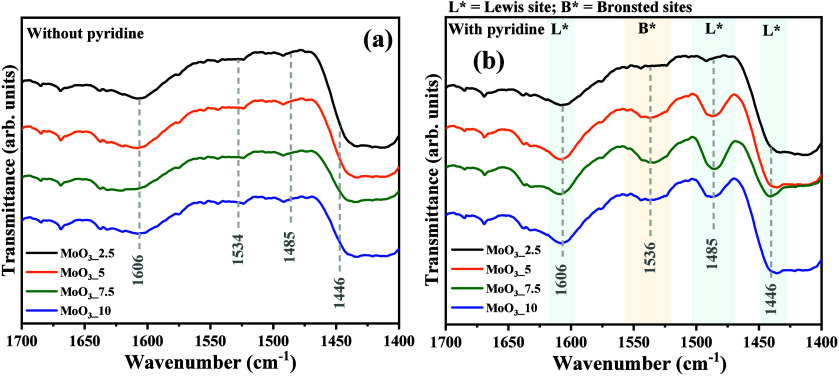
Vibrational FTIR spectrum of MoO_3__2.5, MoO_3__5, MoO_3__7.5, and MoO_3__10 samples (a) before and (b) after the adsorbed pyridine.

As can be seen in Figure 8(a-b), when analyzing the materials using the pyridine probe technique and FTIR analysis to observe bands related to Brønsted and Lewis acidity, significant differences were observed between the spectra with (Figure 8a) and without (Figure 8b) adsorbed pyridine. Therefore, the vibrational spectrum of the MoO_3__2.5 sample did not show any bands related to the pyridine molecule probe in Figure 8(b) between 1400 and 1700 cm^–1^. On the other hand, MoO_3__5, MoO_3__7.5, and MoO_3__10 samples exhibited bands at approximately 1446, 1485, and 1606 cm^–1^, corresponding to the Lewis acid sites, and 1536 cm^–1^, to Brønsted acid sites, demonstrating the presence of acid sites on the catalysts’ surfaces. Based on the structural Rietveld refinement and vibrational Raman spectroscopy, the MoO_3__2.5 sample is essentially composed of molybdenum oxide with hexagonal structure (*h*-MoO_3_); however, the increase of nitric acid in the hydrothermal synthesis shows the obtention of phase mix of hexagonal and orthorhombic structure for MoO_3__5 and MoO_3__7.5 samples, while in the MoO_3__10 sample there was the occurrence of three polymorphs of molybdenum oxide. Thus, following the XPS analysis (described in the following section), it is confirmed that the combination of these polymorphs leads to the appearance of different states of oxidation for molybdenum, in this case, +4, +5, and +6, which can be associated with the oxygen vacancies in the bulk and lattices of the crystal’s structures. Consequently, the Lewis sites are predominantly present in the samples, corroborating the FTIR analysis for pyridine adsorption and confirming the increase in their acidity.

### 3.6 X-ray Photoelectron Spectroscopy (XPS)

**Figure 9:**
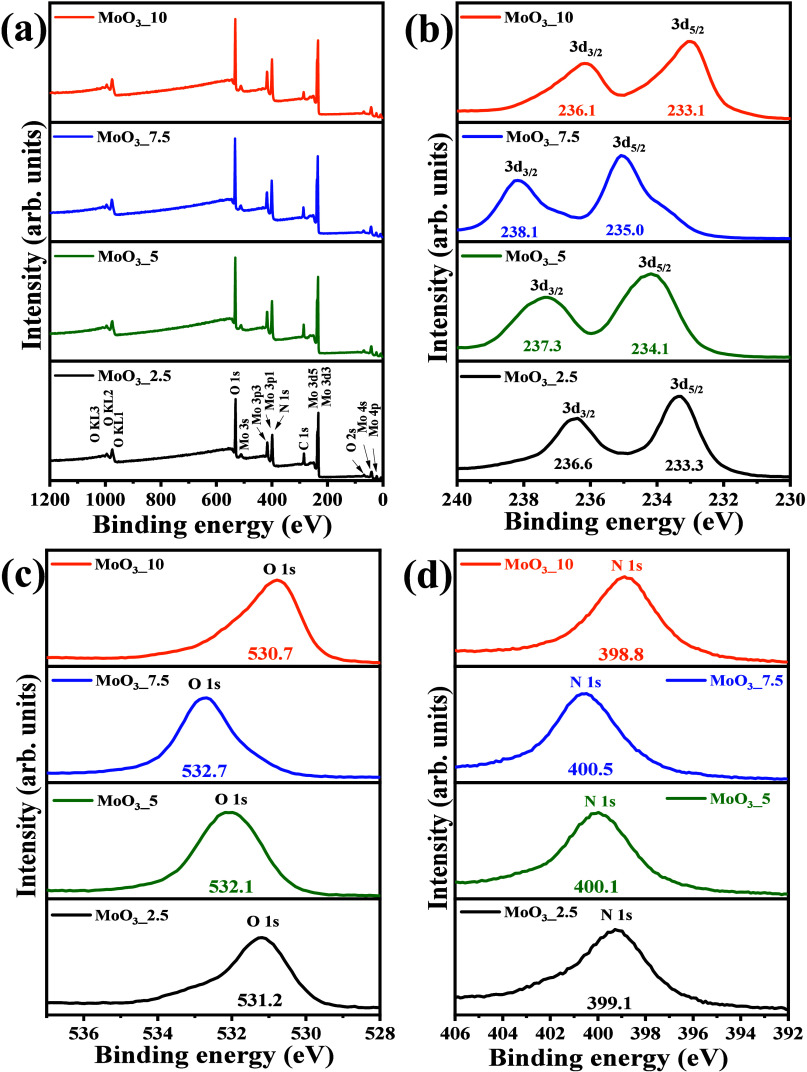
Survey XPS spectrum for (a) MoO_3__2.5, MoO_3__5, MoO_3__7.5, and MoO_3__10 samples and high-resolution XPS spectrum for (b) binding energy of 3d_3/2_ and 3d_5/2_ peaks, (c) O 1s peak, and (d) N 1s peaks for all prepared samples.

Figure 9(d) presents the survey spectra and high-resolution XPS spectra of the binding energy for the Mo 3d_5/2_ and Mo 3d_3/2_ states, as well as the high-resolution spectra for the binding energy of the O 1s and N 1s states, which are present in the composition of the samples MoO_3__2.5, MoO_3__5, MoO_3__7.5, and MoO_3__10. As observed in the survey spectra of the samples, all peaks associated with the states of the molybdenum, oxygen, and nitrogen elements were identified, as well as the peak associated with the C 1s state of carbon, which was used as a standard to calibrate the displacement of the other peaks in the high-resolution spectra.^57^ Therefore, when analyzing Figures 9(b), 9(c), and 9(d), it is possible to note significant variations in the position and profile of the peaks associated with the energy states of the elements present in the samples, corroborating the other characterization techniques performed, which already predicted variations in the composition of the crystalline phases present,^58^ especially regarding the gradual conversion of the hexagonal phase into the orthorhombic phase and, subsequently, the emergence of the monoclinic phase in the MoO_3__10 sample.

Figure S1(a-l), available in the Supporting Information, presents the deconvoluted high-resolution spectra, in this case, of the element’s molybdenum, oxygen, and nitrogen. Thus, it is possible to visualize that the deconvolution of the high-resolution spectra of the Mo 3d_5/2_ and Mo 3d_3/2_ lines in the MoO_3__2.5 sample resulted in significant differences for the position and profile of the Gaussians, with a predominance of the Mo^6+^ state. However, peaks associated with the Mo^5+^ state were also identified. The presence of the Mo^6+^ state indicates the presence of octahedral symmetry clusters [MoO_6_], where the presence of M=O bonds can also occur. On the other hand, the occurrence of Mo^5+^ states indicates the presence of distorted [MoO_5_(OH)] clusters, where the presence of Mo–OH or Mo–O–NH_4_^+^ type bonds can occur for terminal oxygen. This statement corroborates the result of deconvolution of the peak associated with the O 1s state, which resulted in the presence of two bands, one of greater intensity centered at 531.2 eV and another of lesser intensity at 532.8, associated with the oxygens of the crystal lattice shared by the molybdenum atoms in Mo–O–Mo bonds and oxygens coordinated to the ammonium ions, respectively. For the high-resolution spectrum of the N 1s state, three bands appeared, one of greater intensity centered at 398.6 eV and two of lesser intensity at 396.2 eV and 401.8 eV, respectively.^59^ These states are associated with the NH_4_^+^ ions from ammonium heptamolybdate, which is used as a synthesis precursor. For the deconvolution of the high-resolution spectra of the MoO_3__5, MoO_3__7.5, and MoO_3__10 samples, the increase in the Mo^5+^ state was noticeable, as well as the emergence of the Mo^5+^ state, in this case, resulting from the acidic force of the reaction medium, which implied an increase in the Brownian motion, and consequent phase transformation, with the occurrence of a gradual increase in the orthorhombic phase (α-MoO_3_) and monoclinic phase (β-MoO_3_) for the MoO_3__10 sample. This finding implies the presence of a greater density of Lewis acid sites, as already discussed in the analysis by pyridine adsorption, due to the occurrence of tetrahedral symmetry clusters [MoO_4_(OH)_2_], where the 3d_*z*^2^_ and 3d_*x*^2^–*y*^2^_ orbitals are available and favorable sites for interaction with specific substrates, in particular, carboxylic groups in reactions with fatty acids in the presence of alcohols.^60^ Shifts were also observed for the high-resolution spectra of the N 1s and O 1s states of the respective samples, corroborating the predictions made in infrared vibrational spectroscopy that confirmed the significant increase in Lewis sites for the MoO_3__5, MoO_3__7.5, and MoO_3__10 samples. The percentages obtained for the 1s state of nitrogen through deconvolution indicate the presence of nitrogen due to contamination, probably from the carbon tape used as a substrate in data acquisition.

Table 1 summarizes the atomic percentages of the Mo, O, and N elements obtained from the deconvolution of the high-resolution spectra, as shown in Figure S1(a-l). Therefore, it is noted that the atomic ratio between oxygen and molybdenum was 0.79, 0.84, 2.5, and 2.8, respectively. These results indicate a decrease in the hexagonal phase, converting it into the orthorhombic phase, where the proportion between oxygen and molybdenum becomes less influenced by nitrogen.

**Table 1:** Identification, line, binding energy (B.E.), atomic percentage (At.), standard deviation, and O/Mo ratio for a high-resolution deconvoluted spectrum of MoO_3__2.5, MoO_3__5, MoO_3__7.5, and MoO_3__10 samples^a^

ID	Line	B.E. (eV)	At (%)	St. Deviation	O/Mo ratio
**MoO**_**3**_**_2.5**	Mo 3d5/2	233.6	41.57	0.63	0.79
	Mo 3d3/2	236.6			
	O 1s	531.2	33.17	0.07	
	N 1s	399.1	25.25	0.10	
**MoO**_**3**_**_5**	Mo 3d5/2	234.1	40.89	0.52	0.84
	Mo 3d3/2	237.3			
	O 1s	532.1	34.75	0.03	
	N 1s	400.1	24.37	0.12	
**MoO**_**3**_**_7.5**	Mo 3d5/2	235.0	13.64	0.073	2.5
	Mo 3d3/2	238.1			
	O 1s	532.7	38.29	0.09	
	N 1s	400.5	48.08	0.08	
**MoO**_**3**_**_10**	Mo 3d5/2	233.1	13.85	0.72	2.8
	Mo 3d/2	236.1			
	O 1s	530.7	39.34	0.09	
	N 1s	398.8	46.79	0.07	

a **Legend:** ID = Identification; B.E. = Binding Energy; At = atomic percentage obtained through the deconvolution of characteristic XPS B.E. peak.

In the study carried out by Leung et al.^61^ thin films of MoO_3_ were obtained using the nitridation technique in an NH_3_ atmosphere and studied using the XPS technique. The authors concluded, through the deconvolution of the high-resolution spectra of the 3d_5/2_ and 3d_3/2_ states of molybdenum, that the formation of the orthorhombic phase of molybdenum oxide is temperature-dependent, with the occurrence of the Mo^5+^ and Mo^4+^ species. On the other hand, Baltrusaitis et al.^62^ studied the formation of the crystalline phase of molybdenum oxide from the pyrolysis of ammonium heptamolybdate on ITO substrates at 350 °C, followed by additional heat treatment at 500 °C. The authors observed, through the deconvolution of the high-resolution spectrum of the Mo 3d_5/2_ and Mo 3d_3/2_ lines, that the materials obtained exhibit the Mo^6+^, Mo^5+^, and Mo^4+^ states, with molybdenum with oxidation number +5 being predominant.

### 3.7 Optical Properties by UV–vis by Diffuse Reflectance Spectroscopy and Colorimetry

**Figure 10:**
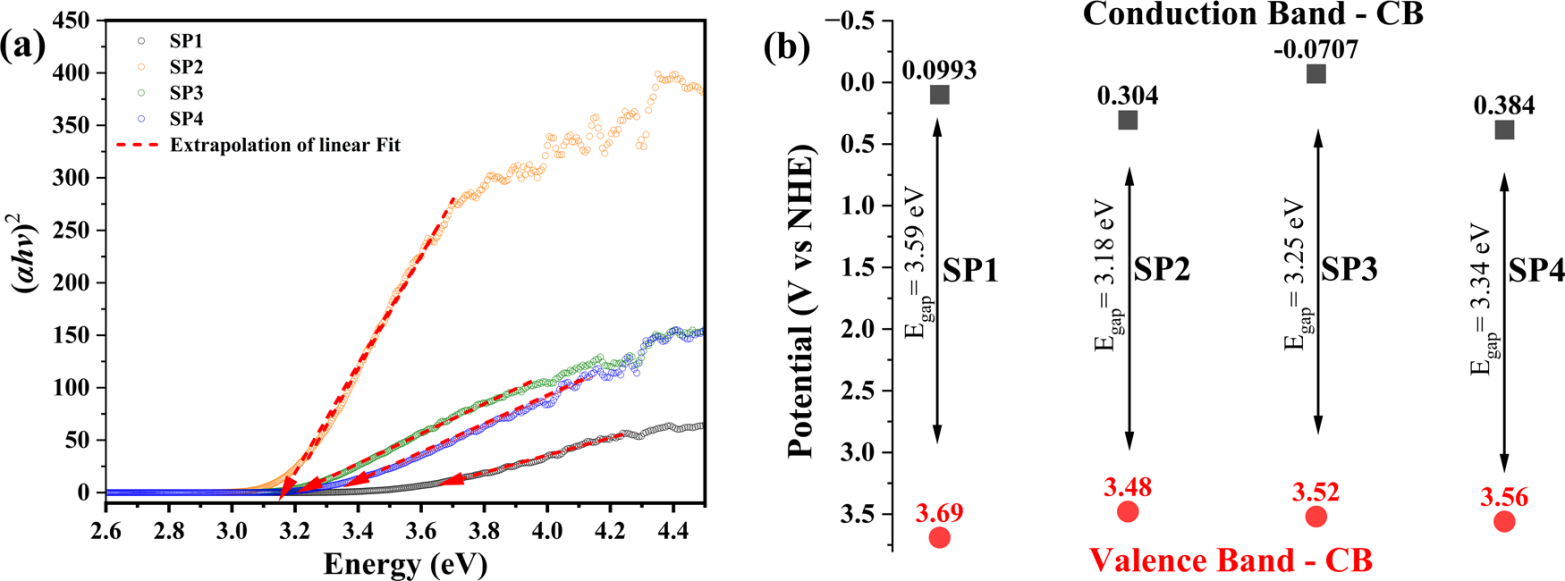
(a) Tauc plot and (b) conduction and valence band position of MoO_3__2.5, MoO_3__5, MoO_3__7.5, and MoO_3__10 samples.

The optical properties of the synthesized materials were investigated by diffuse reflectance UV–vis spectroscopy (UV–vis-DRS) and colorimetric analysis, as presented in Figure 10(a-b) and Table 2. The spectrum presented in Figure 10(a) exhibits strong light absorption at wavelengths higher than 400 nm, the visible spectrum region. The optical bandgap was calculated using the Tauc method, initially converting the percentage reflectance (*R*) data into the Tauc function (α),^63^ dividing the absorption coefficient (*k*) and the scattering coefficient (*s*), which were obtained from eq 4.^64^
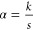
4where the scattering coefficient and the absorption coefficient were obtained by eq 5 and eq 6.
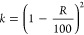
5
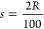
6The wavelength values for the analysis range of the UV–vis spectrum by diffuse reflectance were converted into energy (photons) using Planck’s equation (*E*_*phot*_ = 1240/λ).^29^ Therefore, the *E*_gap_ of the materials was obtained using the Tauc model,^63^ as shown in eq 7.
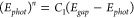
7where *C*_1_ corresponds to the proportionality constant and *n*, the nature of the electronic transition between the orbitals involved in chemical bonds, which give rise to the valence band (VB) and conduction band (CB), respectively. In this case, they can be of the type: *n* = 2 (direct permitted transitions); *n* = 2/3 (direct prohibited transitions prohibited); *n* = 1/2 (indirect permitted transitions); and *n* = 1/3 (indirect prohibited transitions.^65^

**Table 2:**
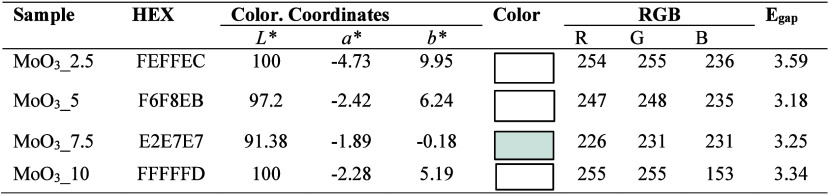
Colorimetric properties of the MoO_3__2.5, MoO_3__5, MoO_3__7.5, and MoO_3__10 samples^a^

a **Legend:** HEX = Color-hex codes; Color. Coordinates = colorimetric coordinates; *E*_gap_ = optical bandgap.

According to the study carried out by Bandaru et al.,^65^ the electronic transitions between the O 2p and Mo 3d orbitals are of the direct permitted type, with the most significant contribution from the O 2p orbitals in the valence band. In contrast, the conduction band is mainly governed by electronic transitions involving the Mo 3d orbitals. The *E*_gap_ value was acquired by extrapolating the paraboloid curve obtained from the plot of (*αE*_*phot*_)^*n*^ vs *E*_*phot*_, where *n* = 2.

As shown in Figure 10(b) and Table 2, the *E*_gap_ values of MoO_3__2.5, MoO_3__5, MoO_3__7.5, and MoO_3__10 samples were 3.69, 3.18, 3.25, and 3.34 eV, respectively. Therefore, it is noted that there was a reduction in the value of *E*_gap_ from 3.69 eV (MoO_3__2.5) to 3.18 eV (MoO_3__5), with the MoO_3__2.5 sample being composed solely of the *h*-MoO_3_ phase, while the MoO_3__5 sample, the mixture of *h*-MoO_3_ and α-MoO_3_ phases, in the proportion of 95.7% and 4.3%, respectively. The same does not happen for samples MoO_3__7.5 and MoO_3__10, observing the increase in the *E*_gap_ value to 3.25 and 3.34 eV, respectively; thus, the decrease in *E*_gap_ is not directly related to the increase in the fraction corresponding to the α-MoO_3_ phase in the composition of the samples.

In the study carried out by Maiti et al.^64^ nanorods composed of *h*-MoO_3_ were efficiently obtained by the epitaxial growth method on fluorine-doped tin oxide (FTO) substrates and silicon substrates - Si (100) and Si (512) under high vacuum, where *E*_gap_ values between 3.17 and 3.38 eV. On the other hand, Ijeh et al.,^31^ also studying molybdenum oxide films, observed an *E*_gap_ value equal to 3.44 eV for pure MoO_3_, indexed to the orthorhombic phase. In this way, it is confirmed that the values obtained in the present study are consistent with those reported in the literature consulted.^30,31,49,64,65^

Using eqs 8 and 9, the energy associated with the position of the valence (*E*_vb_) and conduction (*E*_cb_) bands was determined from the bandgap values obtained by the Tauc method.
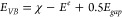
9
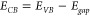
8where *E*^*e*^ is the energy of the free electron (*E*^*e*^ = 4.5 eV) and χ is the electronegativity of MoO_3_. In this case, calculated using eq 10, χ(O) and χ(Mo) are 7.54 and 3.90 eV, respectively.
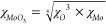
10As shown in Figure 10(b), the *E*_CB_ values for the MoO_3__2.5, MoO_3__5, MoO_3__7.5, and MoO_3__10 samples are 0.0993, 0.304, −0.0707, and 0.384 eV, respectively. Meanwhile, for *E*_VB_, they were 3.69, 3.48, 3.52, and 3.34 eV. These results indicate the semiconductor character of MoO_3_, pure or comprising a mixture of phases (hexagonal and orthorhombic) with characteristics of an n-type semiconductor, that is, an electron donor in a conjugated system. Furthermore, it is suggested that the reduction in *E*_gap_ observed for this sample is related to the depletation layer composed of the heterojunction of structures composed of the *h*-MoO_3_ and α-MoO_3_ phases, which introduces intermediate levels between VB and CB, making excitation/recombination of electrons at a lower energy cost.^33,66^

The colorimetric analysis of the materials, presented in Table 2, was carried out to investigate the characteristic color pattern of each sample obtained. In this case, this is directly related to the increase in acidity of the solution used in the preparation of MoO_3__2.5, MoO_3__5, MoO_3__7.5, and MoO_3__10 samples, being, respectively, 0.6, 1.10, 1.60, and 2.10 mol L^–1^. Therefore, colorimetry is based on investigating the variation of the tristimulus pattern associated with the change in the variables a*, b*, and L*, which indicate the change in the color pattern from red (+a) to green (−a), or from yellow (+b) toward blue (−b), or variation in luminosity (*L*), starting from the lower limit, i.e., 0 to the upper limit 100, thus highlighting very dark and opaque samples, respectively.

As can be seen in Table 2, it is possible to notice the variation in the color pattern of the materials, with different values of the colorimetric coordinates, because of the electronic transitions between the orbitals, which are affected by the presence and type of ligands to the metal centers, type of coordination, size, and morphology of crystals, and synthesis methods. For sample MoO_3__2.5, which displays the hexagonal phase as the only one present in its composition, the color pattern displays maximum luminance value (L*) and coordinate values a* and b*, which is classified as pale-yellow material. It has a strong absorption of photons in the ultraviolet region, associated with an *E*_gap_ of 3.59 eV.

The increased acidity of the reaction medium to obtain the samples MoO_3__5 and MoO3_7.5 resulted in light grayish-yellow (F6F8EB) and light grayish-cyan (E2E7E7) colors, respectively. For sample MoO_3__10, the colorimetric coordinates indicate the formation of a pale-yellow material with HEX and *E*_gap_ codes equal to FFFFFD and 3.34 eV, respectively. The behavior observed for the color pattern is directly related to the mixture of phases for molybdenum oxide, as already confirmed by X-ray diffraction and Raman spectroscopy techniques, which can be related to the presence of the NH_4_^+^ and OH groups in the terminal connections of the [Mo–O] units present in the structure. In the study carried out by Mizushima et al.,^67^ samples of commercial MoO_3_ were treated with nitric acid, obtaining different color patterns depending on the conversion of the alpha and beta phases of MoO_3_.

### 3.8 Catalytic Performance of Samples as Solid Catalysts in the Esterification of Oleic Acid

**Figure 11:**
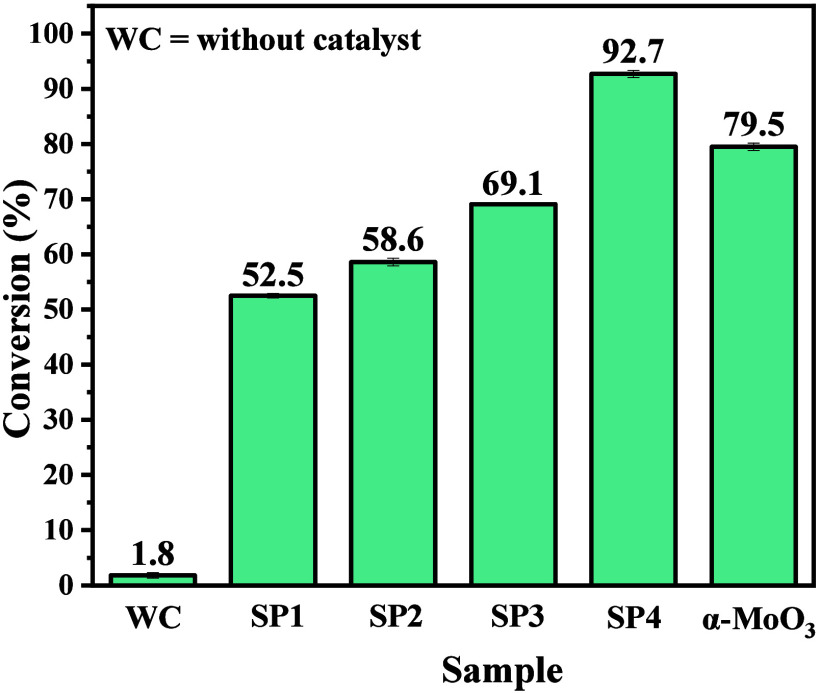
Catalytic performance of MoO_3__2.5 (SP1), MoO_3__5 (SP2), MoO_3__7.5 (SP3), and MoO_3__10 (SP4) samples over the conversion of oleic acid to methyl oleate. The bare α-MoO_3_ synthesized by Pinto et al.^41^ was used as a catalyst for comparison.

Figure 11 shows the catalytic performance of the samples synthesized as solid catalysts in the oleic acid esterification reaction. For comparison purposes, the catalytic activity of a molybdenum trioxide sample was synthesized according to the study carried out by Pinto et al.,^41^ composed of a bare α-MoO_3_ phase. Based on the data obtained, it is possible to see that the esterification reaction in the absence of a catalyst results in a low level of conversion of oleic acid into methyl oleate, in this case, obtaining only 1.8% conversion. According to the literature,^28,39,41,51,68^ this is associated with the energetic barrier (activation energy) for reactivity between fatty acid molecules in the presence of alcohols, mainly long-chain alcohols. However, when using the molybdenum oxide samples synthesized in this study, it was possible to observe a significant increase in conversion percentages, which were between 50.7 and 90.9%, with the lower value associated with sample SP1, while the best performance was for the SP4 sample.

Therefore, it is possible to note that the activity of the obtained catalysts is characterized by a gradual growth of the alpha phase of molybdenum trioxide in the material’s composition. However, when the catalyst described as α-MoO_3_ was used, a conversion equivalent to or greater than the MoO_3__10 sample was not obtained.

This observation makes it possible to confirm that the mixture between the hexagonal, orthorhombic, and monoclinic phases, mainly for sample SP4, leads to an improvement in catalytic properties, with a synergistic effect between the structures, which add optical, textural (10.273 m^2^/g), and catalytic properties of obtained samples. These results agree with the FTIR analysis of pyridine adsorption, where increasing the Lewis sites in the sample MoO_3__10 improves the catalytic performance in the esterification of oleic acid, which agrees with the work done by Silva et al.^14^

**Figure 12:**
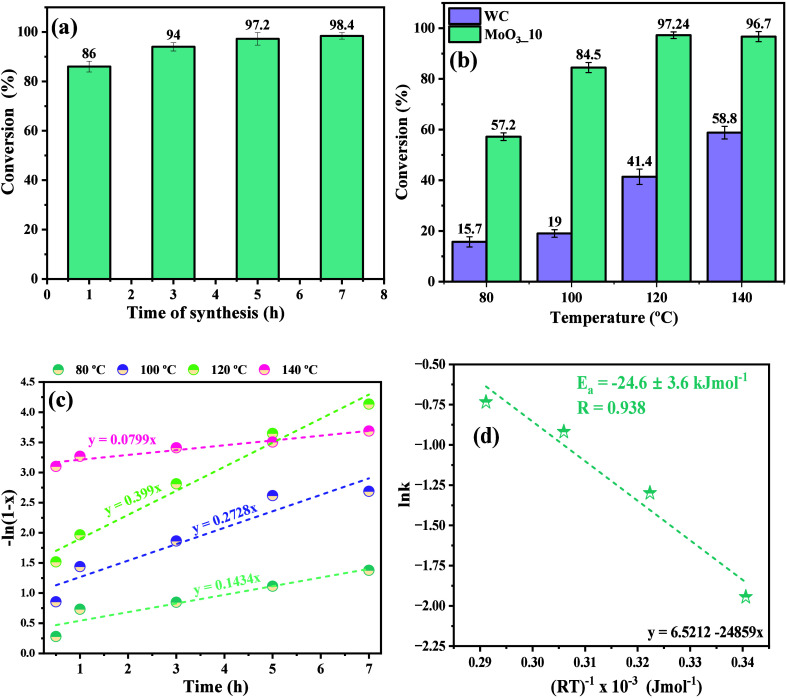
Dependence of conversion percentage for OA in MO at different (a) reaction time, (b) temperature, (c) plot for −ln(1 – *x*), and (d) plot of ln *k* against 1/*RT* (first-order reaction).

Considering that the SP4 catalyst showed a higher yield in the esterification of oleic acid, this sample was used to carry out the study to optimize the reaction conditions. Thus, in Figure 12(a-d), the catalytic activity of the MoO_3__10 sample is presented under varying times and temperatures (80, 100, 120, and 140 °C), adopted in the catalytic experiments. Based on these results, a kinetic and thermodynamic study of the reaction was also carried out.

Based on the information presented in Figure 12(a), increasing the reaction synthesis time leads to an increase in the conversion percentage, obtaining 86% conversion for the process carried out in 1 h. In contrast, for times of 3, 5, and 7 h, 94, 97.2, and 98.4% conversions were obtained, respectively. These results indicate the dependence on time in the conversion process, a result of the system homogenization process, and the frequency of collisions between the reactants, assisted by the catalyst, leading to a lower energy barrier for the conversion into products. Therefore, due to the slight variation in the percentage of conversion obtained for times longer than 3 h, it was decided to use this synthesis time (3 h) in the catalytic tests involving the variation of the other studied factors.

The results of the conversion of oleic acid into methyl oleate under temperature variation are shown in Figure 12(b). Analysis of the results presented in Figure 12(b) makes it possible to confirm that the reactions conducted in the absence of a catalyst did not result in significant conversion percentages. This is due to the energetic barrier related to activation energy (*E*_a_), which is necessary to reach the transition state and, subsequently, conversion into the products of interest, in this case, water molecules and methyl oleate.

In the study reported by Deus et al.,^15^ the optimization of the oleic acid esterification process was investigated using factorial planning in the absence of a catalyst, with variables such as temperature (323, 333, and 343 K), flow of oil injected into the system (1.3, 2.6, and 3.9 g min^–1^), and system pressure (50, 100, and 150 kPa). The results presented by the authors reveal that the best conversion performance of oleic acid into methyl oleate was achieved for the values of temperature, pressure, and flow, corresponding to 343 K, 150 kPa, and 1.3 g min^–1^, respectively. Furthermore, although all of the factors investigated were significant, according to the Pareto chart analysis reported by the authors, the system temperature was the most significant factor among all of the factors investigated. In addition, the activation energy and the pre-exponential factor (*A*_0_) calculated for the process were 59.06 kJ mol^–1^ and 6.51 × 10^6^ L mol^–1^ s^–1^, respectively.

Therefore, corroborating the literature,^69^ an increase in conversion percentage with increasing temperature is evident, where reactions processed at 80, 100, 120, and 140 °C resulted in conversion percentages of 15.7, 19, 41.4, and 58.8%, respectively. However, the addition of MoO_3__2.10 sample as a catalyst for the reaction process increased the conversion, except for the temperature of 140 °C, obtaining percentages of 57.2, 84.5, 97.24, and 97.7%, respectively, at 80, 100, 120, and 140 °C. In this context, based on the literature,^70,71^ it is suggested that the decrease in catalytic performance at a temperature of 140 °C is due to the chemical shift effect of the reaction, which results in the process’s reversibility.

Based on the reaction involved in the production of methyl oleate and water, using the reagents oleic acid - OA (C_18_H_34_O_2_) and methyl alcohol – MA (CH_3_OH), as shown in eq 11, it is possible to estimate the reaction ratio (*r*) as being equivalent to the reaction speed (d[OA]/d*t*), complying with the literature,^15^ first-order reaction kinetics (α = 1). In this case, *k* corresponds to the reaction rate constant, as shown in eqs 12 and 13.
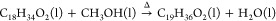
11
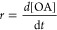
12
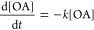
13From eq 13, it is possible to obtain the equation in the form presented in eq 14, where, applying the integral on both sides of the expression (eq 15), the relationship presented in eq 16 is obtained.^69^
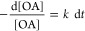
14
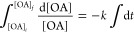
15
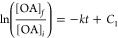
16It is known that the final concentration of OA is equivalent to the product of the initial concentration by the conversion rate - Cr (Cr = 1 – *x*’), where *x*’ is the fractional conversion ratio of oleic acid to methyl oleate. As shown in eq 17, it is possible to rearrange the expression to the form presented in eq 18 and then insert it into eq 19, thus obtaining the expression presented in eq 20. Thus, the expression presented in eq 18 is determined by the reaction rate constant (*k*) and by the slope of the plot of −ln(1 – *x*) against *t*.^15,69^
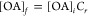
17
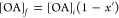
18
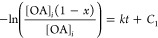
19
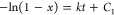
20From the conversion rate data as a function of catalysis time, by the variation in reaction time, it was possible to obtain the plot −ln(1 – *x*’) versus the variation in reaction time (*t*), as shown in Figure 12(c). Therefore, the rate constants for reactions conducted at temperatures of 80, 100, 120, and 140 °C were 14.34 × 10^–2^, 27.82 × 10^–2^, 39.3 × 10^–2^, and 7.99 × 10^–2^ h^–1^. The activation energy by Arrhenius eq (eq 21), enthalpy variation (Δ*H*), Gibbs free energy variation (Δ*G*), and entropy variation (Δ*S*) of the process were calculated using eqs 21, 22, 23, and 24, respectively.^72^
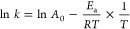
21
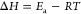
22
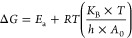
23
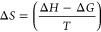
24where *R*, *T*, *K*_B_, *h*, and *A*_0_ are the ideal gas constant (*R* = 8.314 J·K^–1^ mol^–1^), the absolute temperature (K), Boltzmann constant (*K*_B_ = 1.3807 × 10^–23^ J K^–1^), Planck’s constant (*h* = 6.626 × 10^–34^ J s), and pre-exponential factor, respectively.^73^ Thus, the value obtained for the activation energy by plotting ln *k* versus 1/*RT* was 24.6 ± 3.6 kJ mol^–1^. In a previous study,^71^ we reported a value for the activation energy of oleic acid equal to 43.6 kJ mol^–1^ using similar experimental conditions. Therefore, it is confirmed that using the catalyst in the reaction process decreased the *E*_a_ value by approximately 57.1%, as shown in Figure 12(d). Consequently, the energy barrier was reduced, accompanied by an increase in the rate of conversion of the reactants into products.

In Table 3, the values obtained for the thermodynamic variables Δ*H*, Δ*G*, and Δ*S* are summarized as well as the values of *E*_a_ and *A*_0_ obtained in the kinetic-thermodynamic study for the conversion of oleic acid into methyl oleate in the temperatures of 80 °C (253.15 K), 100 °C (373.15 K), 120 °C (393.15 K), and 140 °C (413.15 K), respectively. Based on the results presented, it is noted that the values obtained for enthalpy variation confirm the endothermic enthalpy nature, that is, the need to add heat for the reaction to occur, corroborating the high activation energy associated with the conversion of reactants into products.^51,69,73^ According to the values obtained for the Gibbs free energy (Δ*G*), the increase in the catalysis temperature caused an increase in the spontaneity of the reaction, obtaining values of Δ*G* in the ranges of −67.4 and −78.1 kJ mol^–1^, in this case, for the respective temperatures of 353.15 K (80 °C) and 393.15 K (140 °C). Furthermore, the variation in entropy calculated was between 0.252 and 0.253 kJ mol^–1^, corroborating the reaction’s spontaneity.

**Table 3:** Thermodynamic parameters for esterification of OA over the MoO_3__2.10 sample as a catalyst

Temperature (K)	Δ*H* (kJ mol^–1^)	Δ*G* (kJ mol^–1^)	Δ*S* (kJ mol^–1^)	*A*_0_ (s^–1^)	*E*_a_ (kJ mol^–1^)	*R*^2^
**353.15**	21.6	–67.3	0.252	0.188	24.6	0.937
**373.15**	21.5	–72.7	0.252			
**393.15**	21.3	–78.1	0.253			
**413.15**	21.1	–83.4	0.253			

**Figure 13:**
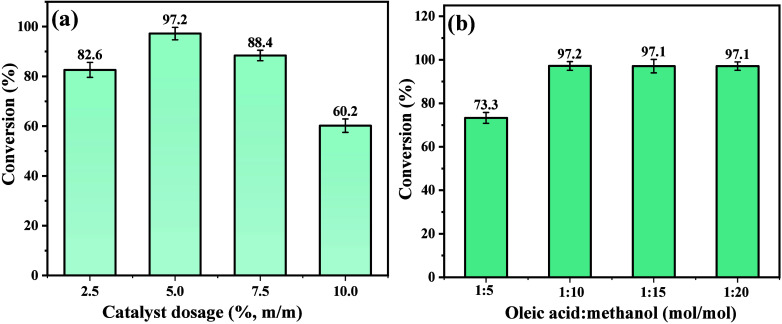
Dependence of (a) catalyst dosage and (b) proportion of OA/MA (mol/mol) in the conversion percentage of methyl oleate.

The influence of the catalyst dosage on the reaction medium, as well as the oleic acid (OA) and methanol alcohol (MA) ratio, was investigated, as can be seen in Figure 13(a-b). Therefore, the variation in the catalyst mass (dosage), in the amounts of 2.5, 5, 7.5, and 10% (m/m), concerning the OA mass, increased by 14.6% of conversion, increasing the catalyst mass from 2.5 to 5%. However, for values above 5%, there was a gradual decrease in catalytic performance, due to the decrease in mass transfer. The decrease in catalytic performance can be associated with the effective collisions between the reactants; in addition, the increase in matter reduces the homogeneity of the system due to the magnetic agitation of the phases present.^1^ Therefore, a dosage of 5% is optimal for the catalytic process. In the study carried out by Cantika, Zulfikar, and Rusli^74^ the decrease in the catalytic performance of ethylenediamine-modified chitosan in the transesterification of palm oil was observed for dosages greater than 0.75 g of the catalyst in the reaction medium. This behavior was attributed to the inefficient interaction of reactant molecules with the catalyst surface, resulting in unstable reaction intermediates and, consequently, the reversibility of the process.

On the other hand, when investigating the proportion between the reactants, that is, oleic acid and methanol, in the proportions of 1:5, 1:10, 1:15, and 1:20 (mol/mol), it was noted that there was no significant increase in proportions greater than 1:10, which stabilized at a conversion percentage close to 97.2%. These results confirm the lower cost related to the reaction process when using the catalyst studied, compared to those described in the literature,^1,11,12,14,15,41,72^ which is due to the reduction in the amount of alcohol used in the esterification of oleic acid. The reduction in the conversion percentage for proportions greater than 1:10 indicates the occurrence of the reversibility effect of the reaction.

**Figure 14:**
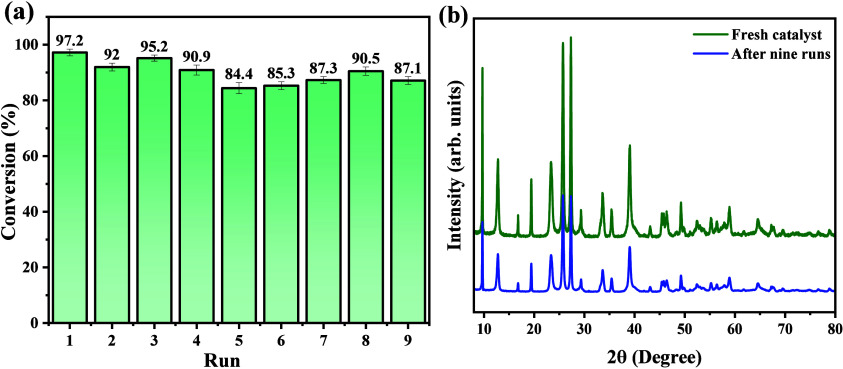
(a) Reusability and (b) the XRD diffraction pattern of the MoO_3__2.10 sample after the ninth run.

The ability to reuse the MoO_3__2.10 sample as a catalyst in the oleic acid esterification reaction and its stability in different catalytic cycles was investigated in nine consecutive catalytic tests, as shown in Figure 14(a,b). After each experiment, the catalyst was collected, washed with hexane to remove organic fractions on its surface, and used in the next cycle. Therefore, supported by the results presented, the reuse capacity of the catalyst is confirmed, which showed a decrease of 10.1% at the end of the ninth catalytic cycle. Furthermore, it presented high chemical stability, confirmed by the profile obtained for the diffraction pattern, as shown in Figure 14(b), in which all crystallographic planes are indexed as characteristic of the phase mixture of MoO_3_, with minor changes in the intensity of diffraction peaks. Therefore, the high stability of samples was suggested after nine consecutive catalytic cycles.

## Conclusion

Applying the hydrothermal method, it was possible to efficiently synthesize molybdenum oxide microcrystals with a hexagonal and orthorhombic structure at 160 °C, under the influence of nitric acid concentration. The materials obtained were characterized by X-ray diffraction (XRD) and Raman spectroscopy, which confirmed the formation of the hexagonal phase for MoO_3_ at a concentration of 0.6 mol L^–1^ (MoO_3__2.5). On the other hand, the addition of nitric acid at concentrations of 1.10 mol L^–1^ (MoO_3__5), 1.60 mol L^–1^ (MoO_3__7.5), and 2.10 mol L^–1^ (MoO_3__10), where the percentage of the orthorhombic phase, that is the alpha phase, was 72.78%. In contrast, the hexagonal phase was 9.38% and 17.84% for the beta phase for the concentration of 2.10 mol L^–1^. Furthermore, all vibrational modes for the structure were identified in vibrational Raman and infrared spectroscopy, corroborating to X-ray diffraction analysis. The images collected by scanning electron microscopy revealed the obtainment of microcrystals with hexagonal shapes when using a concentration of 0.6 mol L^–1^, which is already expected in this respective sample. However, increasing the solution concentration resulted in rod-like microcrystals; a characteristic morphology of the orthorhombic phase for MoO_3_ was verified, corroborating the other characterization techniques. The XPS analysis confirms the occurrence of different states for molybdenum in the structures, where the presence of Mo^4+^ and Mo^5+^ is associated with the available Lewis sites on the structure surface, also confirmed by adsorption/desorption of pyridine in the FTIR spectrum, improves their catalytic performance. The catalytic tests revealed the excellent performance of the obtained materials, emphasizing sample MoO_3__10 as a catalyst for the oleic acid esterification reaction to obtain methyl oleate. In this case, obtaining conversion percentages greater than 97%, using a catalyst proportion of 5% (w/w) concerning the mass of oleic acid, oleic acid/methyl alcohol ratio of 1:10 (mol/mol), and temperature of 120 °C. Furthermore, after nine catalytic cycles, it showed a conversion efficiency of 87.1%, confirming the effective reuse capacity of the catalyst.

## Supporting Information Available

The complementary Rietveld refinement results (Table S1) and the High-Resolution deconvoluted XPS spectrum (Figure S1a-l) (PDF)
