# Supplementary material for: The Synergic Effect of h-MoO3, α-MoO3, and β-MoO3 Phase Mixture as a Solid Catalyst to Obtain Methyl Oleate
Source: ACS Appl Mater Interfaces. 2024 Oct 29;16(44):60103–21. doi: 10.1021/acsami.4c08804 (PMC11551912; doi:10.1021/acsami.4c08804)
Supplement: Supplementary file 1 — am4c08804_si_001.pdf [file am4c08804_si_001.pdf]

Supporting Information (SI)

The synergic effect of *h*-MoO_3, α-MoO_3, and β-MoO_3 phase mixture as a solid catalyst to obtain methyl oleate

Gabrielle Sophie Medeiros Leão[1#], Marcos Daniel Silva Ribeiro[1#], Rubens Lucas de Freitas Filho[2], Libertalamar Bilhalva Saraiva[1], Ramón R. Peña-Garcia[3], Ana Paula de Carvalho Teixeira[2], Rochel Montero Lago[2], Flávio Augusto Freitas[4], Silma de Sá Barros[5], Sérgio Duvoisin Junior[6], Yurimiler Leyet Ruiz[7], and Francisco Xavier Nobre[1*]

[1]Departamento de Química, Meio Ambiente e Alimentos (DQA), Grupo de Recursos Energéticos e Nanomateriais (GREEN Group), Instituto Federal de Educação, Ciência e Tecnologia do Amazonas, Campus Manaus Centro, Manaus, 69020-120, AM, Brazil

[2]Departamento de Química, ICEx, Universidade Federal de Minas Gerais, UFMG, Belo Horizonte, MG 31270-901, Brazil.

[3]Universidade Federal Rural de Pernambuco, programa de Pós-Graduação em Engenharia Física

[4]Núcleo de Materiais e Energia – Centro de Bionegócios da Amazônia. Av. Gov. Danilo de Matos Areosa, 160 - Distrito Industrial I, Manaus - AM, 69075-351, Brazil

[5]Programa de Pós-graduação em Engenharia de materiais, Escola de Engenharia de Lorena, Universidade de São Paulo, Estrada Municipal Chiquito de Aquino, nº 1000 – Mondesir, Lorena, SP, 12612-550, Brazil.

[6]Curso de Engenharia Química, Universidade do Estado do Amazonas, Escola Superior de Tecnologia, Av. Darcy Vagas, 1200, Parque Dez de Novembro, Manaus, AM, 69050-020, Brazil

[7]Departamento de Engenharia de Materiais, Laboratório de Processamento de Materiais Tecnológicos (LPMaT), Universidade Federal do Amazonas, Instituto de Ciências Exatas, Rua Av. General Rodrigo Otávio Jordão Ramos, 1200, Coroado I, Manaus, 69067-005, Brazil

[#]These authors have been equally contributed to this manuscript.

**Corresponding author: Francisco X. Nobre (Francisco.nobre@ifam.edu.br)*

Table of content **Page no**

1)Complementary Rietveld Refinement Results **S3**

2) **High Resolution deconvoluted XPS spectrum** **S4**

1. Complementary Rietveld Refinement Results

The complementary study of crystallographic information of samples MoO_3_2.5, MoO_3_5, MoO_3_7.5, and MoO_3_10, is performed out by structural Rietveld refinement, in focus, the lattice parameters, unit cell volume, crystallite size and phase composition.

Table S1: Rietveld refinement results obtained for samples MoO_3_2.5, MoO_3_5, MoO_3_7.5, and MoO_3_10.

Parameters	Samples				ICSD 87962	ICSD 76651	ICSD 80577
	MoO_3_2.5	MoO_3_5	MoO_3_7.5	MoO_3_10			
**h-MoO_3**							
a (Å)	10.575(3)	10.574(3)	10.578(1)	10.468(7)	10.576(1)		
b (Å)	10.575(3)	10.574(3)	10.578(1)	10.468(7)	10.576(1)		
c (Å)	3.725(7)	3.725(7)	3.725(7)	3.708(2)	3.728(1)		
V (Å^3)	360.85(02)	360.78(2)	361.03(9)	351.94(7)	361.12(12)		
$\overline{D}_{hkl}$ (nm)	46	55	56	77			
Xr (%)	100	97.96	95.71	9.38			
**α-MoO_3**							
a (Å)		13.855(9)	13.867(3)	13.758(5)		13.8550	
b (Å)		3.696(5)	3.6949(4)	3.672(7)		3.7010	
c (Å)		3.959(7)	3.957(7)	3.940(1)		3.9620	
V (Å^3)		202.81(1)	202.78(1)	199.09(9)		203.16	
$\overline{D}_{hkl}$ (nm)		22	21	20			
Xr (%)		2.04	4.29	73.78			
**β-MoO_3**							
a (Å)				3.936(5)			3.954(1)
b (Å)				3.670(2)			3.687(2)
c (Å)				7.078(3)			7.095(4)
V (Å^3)				99.38(9)			100.47(8)
$\overline{D}_{hkl}$ (nm)				120			
Xr (%)				16.84			

Legend: V = Unit cell volume; $\overline{D}_{hkl}$ = crystallite size and X_r = phase composition.

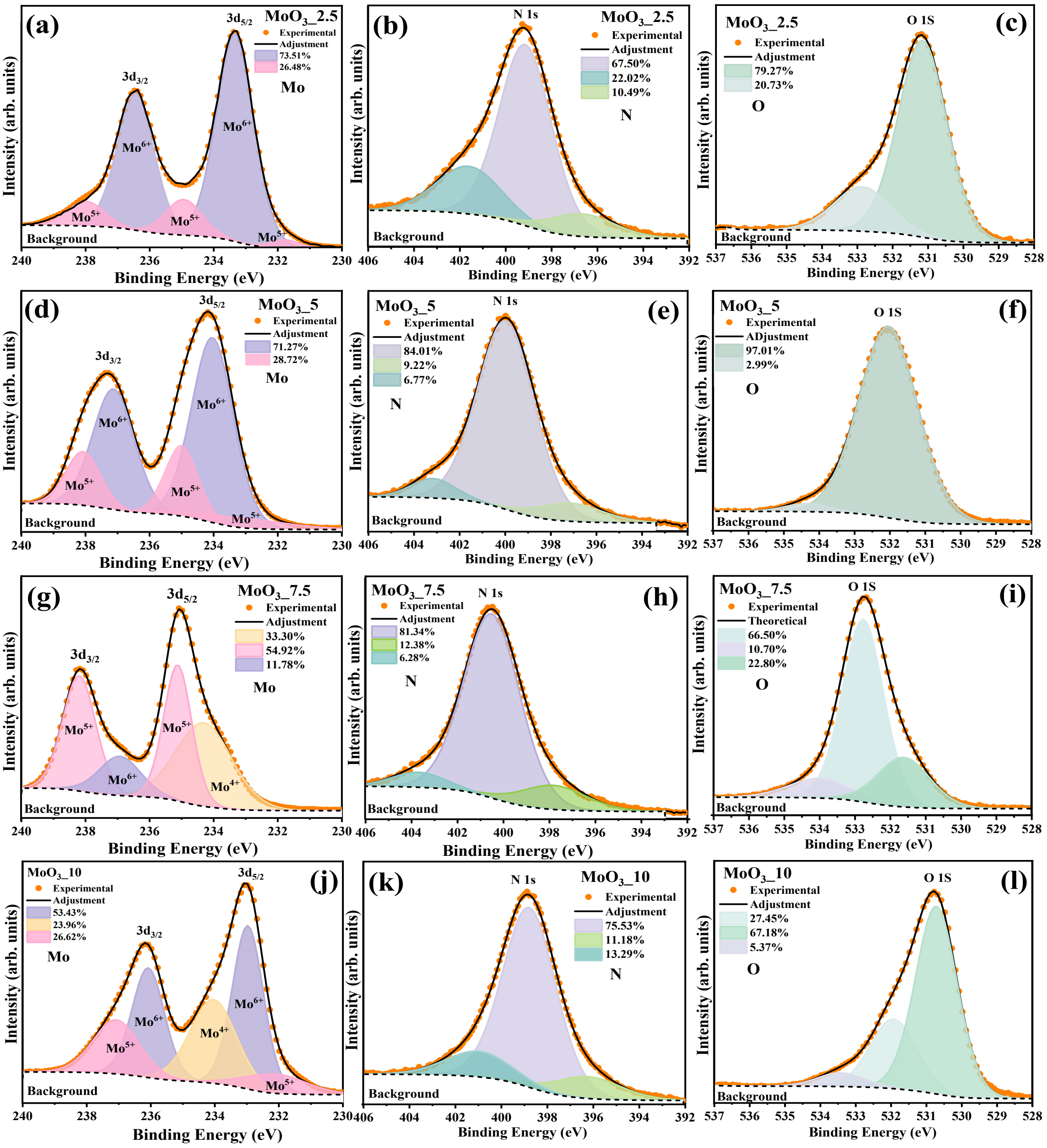

Figure S1: Deconvoluted high resolution Mo $3d_{3/2}$ and $3d_{5/2}$, O 1s and N 1s XPs spectrum of Rietveld refinement results obtained for samples (a-c) MoO_3_2.5, (d-f) MoO_3_5, (g-i) MoO_3_7.5, and (j-l)MoO_3_10.
